# Supplementary material for: 2.6-Å resolution cryo-EM structure of a class Ia ribonucleotide reductase trapped with mechanism-based inhibitor N3CDP
Source: bioRxiv. 2024 Oct 10:2024.10.09.617422. Preprint. [Version 1] doi: 10.1101/2024.10.09.617422 (PMC11482829; doi:10.1101/2024.10.09.617422)
Supplement: Supplement 1 [file media-1.pdf]

Supporting Information for

2.6-Å resolution cryo-EM structure of a class Ia ribonucleotide reductase trapped with mechanism-based inhibitor N_3CDP

Dana E. Westmoreland[2†], Patricia R. Feliciano[1,2,3, †], Gyunghoon Kang[1,2,3], Chang Cui[3,4], Albert Kim[3], JoAnne Stubbe[2,3], Daniel G. Nocera[4], and Catherine L. Drennan[1,2,3*]

[1]Howard Hughes Medical Institute, Massachusetts Institute of Technology, Cambridge, MA 02139.
[2]Department of Biology, Massachusetts Institute of Technology, Cambridge, MA 02139.
[3]Department of Chemistry, Massachusetts Institute of Technology, Cambridge, MA 02139.
[4]Department of Chemistry and Chemical Biology, Harvard University, Cambridge, MA 02138

[†]Authors contributed equally.

*Catherine L. Drennan

Email: cdrennan@mit.edu

This PDF file includes:

Supplementary Material and Methods
Figures S1 to S18
Tables S1 to S16
SI References

Supplementary Materials and Methods

Materials

For protein expression, purification, and spectroscopical expriments, Luria broth, phenylmethylsulfonyl fluoride (PMSF), 4-(2-hydroxyethyl)-1-piperazineethanesulfonic acid (HEPES), $MgSO_4$ and β-Mercaptoethanolwere purchased from Sigma-Aldrich; isopropyl β-d-1-thiogalactopyranoside (IPTG), L-arabinose, kanamycin (Km), ampicillin (Amp), (hydroxymethyl)aminomethane (Tris), and dithiothreitol (DTT) were purchased from GoldBio; imidazole, glycerol, and EDTA were obtained from Oakwood Chemicals; DEAE, Q-sepharose, Sephadex G-25 resins were obtained from GE Healthcare Life Sciences; Ni-NTA agarose resin was purchased from Qiagen. For the Cryo-EM grids preparation, deoxyadenosine triphosphate (dATP) and adenosine triphosphate (ATP) were purchased from Sigma-Aldrich. HEPES buffer, $MgSO_4$, and EDTA were purchased from Sigma-Aldrich.

Protein preparation

The α2 and β2 subunits of *Escherichia coli* class Ia ribonucleotide reductase (RNR) were prepared as previously described (1, 2). The protein concentrations were determined using the extinction coefficients at 280 nm of 189 mM^{-1} cm^{-1} for the α2 dimeric subunit and 131 mM^{-1} cm^{-1} for the β2 dimeric subunit (3).

Synthesis of 2'-azido-2'-deoxycytidine-5'-diphosphate (N_3CDP)

The 2′-azido-2′-deoxycytidine-5′-diphosphate (N_3CDP) was synthesized by myosin cleavage of 2′-azido-2′-deoxycytidine-5′-triphosphate (N_3CTP) using an adaption of a previous protocol (4). N_3CTP was purchased from Trilink Biotechnologies and myosin from rabbit muscle was purchased from Sigma-Aldrich. To synthesize N_3CDP, 1 mM N_3CTP was treated with 0.5 units of myosin in digestion buffer (25 mM potassium borate, pH 9, 5 mM $CaCl_2$, 50 mM KCl) at 25 °C for 30 min. Myosin was then removed by centrifugation using an Ultracel-10 KDa centrifugal filter (EMD Millipore). The filter was washed twice with 250 µL digestion buffer to maximize recovery. The flow-through was combined and loaded onto a DEAE Sephadex A-25 column (15 mL column volume) pre-equilibrated with 150 mL of 50 mM triethylammonium bicarbonate (TEAB) buffer (pH 7.6) at 4°C. The column was washed with 200 mL of 50 mM TEAB buffer and eluted with a linear gradient of 50-600 mM TEAB buffer (150 mL each buffer; pH 7.6). Fractions of 4 mL were collected and the fractions with absorption at 271 nm were combined and lyophilized. The product was redissolved in water and characterized by ^{1}H and ^{31}P nuclear magnetic resonance spectroscopy. The final concentration of N_3CDP in solution was determined using the extinction coefficient at 271 nm of 9100 M^{-1} cm^{-1}.

E. coli class Ia RNR reaction with N_3CDP

The reaction mixture containing 0.75 µM wt-α2, 3 mM ATP, 25 µM dATP and 0.2 mM N_3CDP in assay buffer (50 mM HEPES, 15 mM $MgSO_4$, 1 mM EDTA, pH 7.6) was incubated in a 25 °C circulating water bath for 1 min. The total volume was 200 µL. The reaction was initiated by the addition of 1.5 µM wt-β2, immediately transferred to an electron paramagnetic resonance (EPR) spectroscopy tube in a 25 °C circulating water bath and manually quenched in liquid isopentane cooled by liquid nitrogen after 45 s. The EPR spectra were collected at 77 K on a Bruker EMX X-band spectrometer equipped with a liquid helium cryostat. The spectra (**Fig. S3**) were measured with a 9.4 GHz microwave frequency, a 32 µW power, a 1 G modulation amplitude, a 100 kHz modulation frequency, a 30 s scan time, and averaged over 128 scans.

Cryo-EM grid preparation

Quantifoil R2/2 Cu 300 mesh grids were glow-discharged at -15 mA for 60 s using a PELCO easiGlow cleaning system and subsequently used for cryo-EM grid preparation. To promote the formation of the α2β2 complex, 0.75 µM α2 was incubated with nucleotides (25 µM dATP, 3 mM ATP, and 0.2 mM N_3CDP) in assay buffer (50 mM HEPES pH 7.6, 15 mM $MgSO_4$, and 1 mM EDTA) for 2 min at room temperature. Following incubation, 1.5 µM β2 (2-fold excess to maximize complex formation) was added to initiate the reaction and the mixture was incubated for an additional 45 s

at room temperature. The reaction mixture was then applied to the mesh grid (3 μL) and blotted for 5 s (Whatman filter paper #1) at 10 °C and 95 % humidity. The grid was then plunged in liquid ethane using a Thermo Fisher Scientific Vitrobot (Mk IV) cryo-plunger. Grids were stored in liquid nitrogen until data collection. The final protein solution contained 0.75 μM α2, 1.5 μM β2, 25 μM dATP, 3 mM ATP, 0.2 mM N_3CDP, 50 mM HEPES, pH 7.6, 15 mM $MgSO_4$, and 1 mM EDTA.

Cryo-EM data collection

Cryo-EM data collection was performed at the Cryo-EM Core Facility at the University of Massachusetts Medical School at Worcester using a Thermo Fisher Titan Krios 300 kV electron microscope equipped with a Gatan GIF K2 camera. The data collection parameters used were a defocus range of -1 to -2.3 μm, magnification of 105,000 X, with two shots per hole and pixel size of 0.415 Å (super-resolution, unbinned), 30 frames, and 1.6982 $e^-/Å^2$/frame dose. The collected data set contained 6,007 movies. The cryo-EM data collection statistics are summarized in **Table S1**.

Cryogenic-electron microscopy data processing.

The frames of each dose-fractionated image stack were corrected for beam-induced motion using MotionCor2 (5) within the RELION-4.0 software suite (6). The contrast transfer function (CTF) parameters of each motion-corrected micrograph were estimated using CTFFIND 4.1 (7) implemented in the RELION-4.0 software suite (6). Micrographs with a CTF fit resolution greater than 5 Å were discarded from further analysis. Based on this parameter, the data set containing 6,007 movies was truncated to 5,973 micrographs. These micrographs were used for particle selection using Topaz (8) implemented within the RELION-4.0 software suite (6). Initially, 6199 particles were automatically picked from a randomly generated subset of 20 micrographs using Laplacian-of-Gaussian picking within RELION-4.0 (particle diameter range 150 – 190 Å, box size of 576 pix downscaled to 288 pix). The automatically picked particles were classified into 50 2D class averages to exclude non-particle images from Topaz training. Nine of these 2D classes containing 2070 particles were used to train a neural network automated pipeline for particle picking in Topaz within the RELION-4.0 suite, which picked 1,029,442 particles (particle diameter of 170 pix, box size of 576 pix downscaled to 288 pix) from the 5,973 micrographs using a cut-off of -2.25 for positive signal (**Figure S4**).

These 1,029,442 particles were sorted into 200 2D class averages (mask diameter of 180 Å) (**Figure S4**). Of these, a total of 760,117 α2β2-like particles from 57 2D classes were used to generate an *ab initio* 3D model using no imposed symmetry (i.e. C1) and two classes in RELION-4.0. This set of 760,117 α2β2-like particles was used for 3D classification into four classes (**Figure S4**) using the 558,872-particle class of the two-class *ab initio* 3D model as a reference (**Figure S4**). Class 1 (440,549 particles) displayed an α2β2-like architecture with secondary structure features and the particles from this class following re-extraction at full scale (box size 576 pix) were used for unmasked reference-free 3D auto-refinement yielding a 3.4-Å resolution 3D reconstruction. To further improve map quality, a mask was generated and used to perform post-processing (map sharpening) yielding a 3.1-Å resolution 3D model. Following map sharpening, CTF refinement of the anisotropic magnification followed by the per-particle defocus was performed in RELION-4.0. Bayesian polishing was also performed in RELION-4.0. The refined particle set (440,549 particles) was used to re-run 3D auto-refinement and post-processing, yielding a final reconstruction at 2.6-Å resolution (FSC = 0.143 (**Figures S4** and **S5**)). The local resolution map was estimated using RELION-4.0 (6) (**Figure S6**). The 3D reconstruction parameters and statistics are summarized in **Table S1**.

Model building, refinement, and validation

The refined cryo-EM map was subjected to further sharpening by density modification in Phenix (9) using the two unfiltered half maps from the final round of 3D-refinement, a soft mask, a resolution of 2.6 Å and a molecular weight of 260690.0 Da. The coordinates of the cryo-EM structure of the α2β2 *E. coli* class Ia RNR (PDB 6W4X) (10) were used as the starting model to dock into the final cryo-EM 3D reconstruction using UCSF ChimeraX (11). The model was then fit into the map using phenix.dock_in_map. Since the cryo-EM structure of α2β2 *E. coli* class Ia RNR (PDB 6W4X) (10)

did not have ATP molecules bound in the cone domains, coordinates from a two ATP bound crystal structure of α2 (PDB 8VHN) (28) were used to guide the cone domain model building. The cryo-EM map is of better quality in α than in α′. The α2β2 model was refined by iterative rounds of real-space refinement using phenix.real_space_refine and the initial round of real space refinement included simulated annealing to minimize model bias (9). The geometry restraints for ATP and dATP were generated by phenix.elbow. The cryo-EM map and model were validated using phenix.validation_cryoem (9), and the model quality was evaluated using MolProbity (12). The refinement and model statistics are summarized in **Table S1**. The residues visualized in the structure, of a total of 761 residues for the α subunit and 375 residues for the β subunit, are listed in **Table S2**. Figures were created with UCSF ChimeraX (11). Software packages were compiled by SBGrid (13).

SN-CDP Modeling and Refinement

Bennati and coworkers modeled their spectroscopic data of the nitrogen-based adduct (the "SN-CDP" adduct) formed during N_3CDP inhibition of RNR using density function theory (DFT) (14). This DFT model of the SN-CDP adduct had three simplifications: the 5′ diphosphate group was approximated by a hydroxyl group; the 2′ cytidine base was approximated as a primary amine; and the cysteine residue was approximated as a SCH_3 moiety **(Figure S8)**. Due to these simplifications, we could not exclusively use the DFT coordinates, which we obtained from the Bennati lab, to generate a parameter file for refinement of SN-CDP. Therefore, we generated a parameter file for CDP using phenix.elbow (9) and manually modified it to be consistent with the spectroscopic data and the DTF model. An initial round of real space refinement in phenix without restraints was used to place SN-CDP into the cryo-EM map. A second round of refinement was run restraining the distance between the nonbonded atoms N3′ and C1′ to 3.399 ± 0.010 Å and N3′ and C4′ to 2.425 ± 0.010 Å to be consistent with the N3′-H1′ and N3′-H4′ experimental distances obtained by electron spin echo envelope modulation (ESEEM) spectroscopy (**Fig. S8B, D**) (15, 16). The N3′ to Cys225 S distance was restrained to 1.634 ± 0.050 Å (**Fig. S8B**), consistent with the DFT model, in which Cys225 is approximated by a methyl sulfide (**Fig. S8A,B**) (14). The position of the Cys255 side chain, which is in a standard rotamer conformation, is not the same as the methyl sulfide position (**Fig. S8E**). Modeling of the Cys225 side chain to be positioned like the methyl sulfide, results in a non-standard rotamer for cysteine and places the side chain out of the density. Given the difference between a cysteine side chain and a methyl sulfide, we allowed angles involving the cysteine sulfur, C3′-N3′-S-Cys225 and N3′-S-βC-Cys225, to deviate from the angles for the DTF model with the methyl sulfide substituting for Cys225 (**Fig. S8D**). Given that the DFT calculations indicated that a range of dihedral angles for both S-N3′-C3′-C4′ and βC-S-N3′-C3′ could explain the spectroscopic data, no dihedral restraints were employed. The geometry of the final model of SN-CDP is given in **Fig. S8**, stereoviews of the SN-CDP binding compared to CDP binding is given in **Fig. S9**, and the fit of the SN-CDP adduct to the cryo-EM map is shown in **Fig. 2**.

Water Molecule Modeling

Water molecules were added automatically using Coot (17) at an rmsd level of 1.4 and a distance range of 2.2 – 3.5 Å to account for the average hydrogen bonding distance of water (18). These water molecules were inspected manually and the water molecules that were added to regions with smeared density were removed. Additional waters were added manually throughout the structure based on the following criteria: a density threshold of 0.235; 1.67 sigma and a hydrogen bonding distance of 2.2 – 3.5 Å to a polar residue or another water molecule. The majority of the water molecules identified were previously observed in crystal structures of α2 (19-23) or β2 (22, 24, 25) (see **Tables S3-S6**). Water molecules identified automatically by Coot but not observed in a crystal structure are shown in **Table S7**, and water molecules added manually but not observed in a crystal structure are shown in **Table S8**. To further validate the modeled water molecules, we ran Q-score analysis (26), which indicated an expected Q-score of 0.7674 (out of 1) at 2.6 Å resolution as the value below which water molecules may not be reliably resolvable. 198 of the 674 water molecules were below this expected Q-score. We chose to retain all water molecules below this threshold that had a corresponding water molecule in the crystal structures because visual inspection indicated that the Q-score analysis was under-identifying water molecules that are conserved, as has been

previously observed (26). We re-examined all waters below a Q-score of 0.7674 that did not have a correlate in the known crystal structures and removed 33 waters that had a Q-score of 0.72 or below. For complete transparency, we have included all Q-score values in **Tables S3-S8**. The final number of water molecules in our structure is 624, including the 6 water molecules associated with Mg^{2+} ions. Of the PCET pathway waters, only Water 511 in Chain C (the Fe1-bound water) is significantly below the Q-score threshold and, with the single exception of Water 918 in Chain B, which has been previously observed in three crystal structures (**Table S4**), all remaining PCET pathway waters are above our designated cutoff of 0.72. All water molecules within the described water channels are above this 0.72 cutoff.

Water Channel Analysis

Water channels in α2β2 were identified using Caver 3.0.3 (27). The minimum probe radius was set to 1.5 Å, which is 0.1 Å larger than the Coulombic radius of water, but 0.2 Å smaller than the van der Waals radius of water (28), and the desired radius was set to 2.2 Å, which is the minimum radius of a hydrogen bond to water (29). The shell depth was set to 5 Å, the shell radius was set to 3 Å, and the clustering threshold was 3.5. All protein residues and nucleotides were included in the calculation. For the yellow water channels (**Fig. S16A, B**), the starting point optimization was set to within 4 Å of Y356β. For the pink water channel (**Fig. S16C**), the starting point optimization was set to within 3 Å of the water that forms a 2.2-Å hydrogen bond with Y356β (**Fig. 4D** and **5C**, orange).

Surface Area Analysis

PDBePISA (30) was used to determine the surface area between individual subunit chains by uploading the structural model and selecting the interfaces option in the web interface. For the surface area of β2 buried by the cone domain of α2, residues 96-745 of chain B were manually deleted and the truncated model was uploaded to PDBePISA.

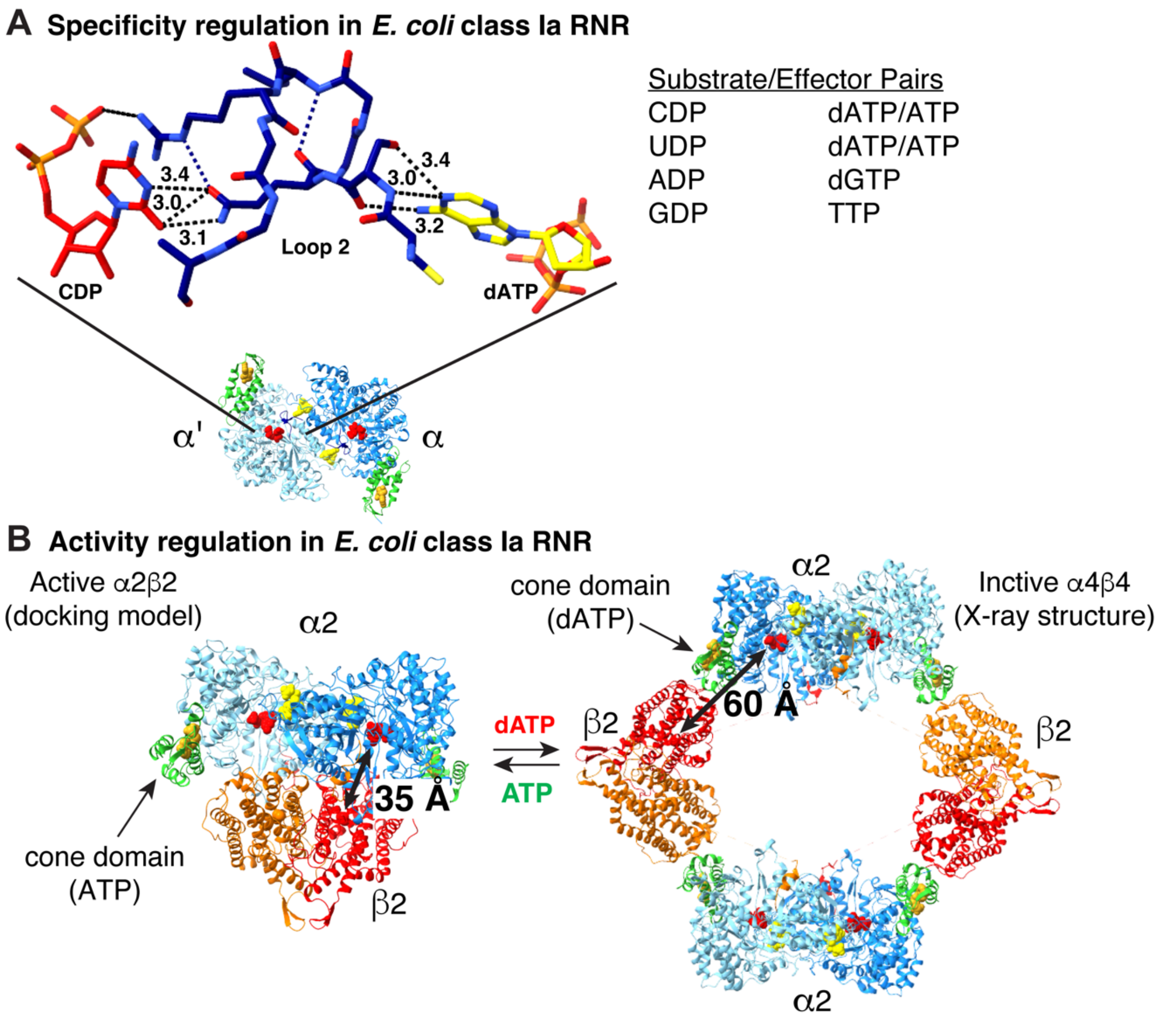

Figure S1. Molecular basis of allosteric regulation of substrate specificity and enzyme activity in *E. coli* class Ia RNR. (A) Preference for the four substrates of class Ia RNR (UDP, CDP, ADP, GDP) is modulated by allosteric specificity effectors (dATP/ATP, GTP, TTP) via a loop at the dimer interface called loop 2. Structure of the α2 subunit of *E. coli* class Ia RNR is shown with substrate (red carbons), specificity effector (yellow carbons), and loop 2 (navy blue carbons). The substrate/effector pairs are listed. **(B)** Overall RNR activity is regulated by allosteric activity effectors dATP (down-regulation) and ATP (up-regulation). At low dATP concentrations, dATP acts predominantly as a specificity effector to promote the reduction of CDP and UDP. At higher dATP concentrations, dATP binds to the activity site in the cone domain (green, residues 5-95) inducing the formation of an α4β4 ring (PDB 5CNS), which is an inactive state of the enzyme. In this ring structure, the radical subunit, β2, is held away from α2 so that the ~35-Å distance that the radical needs to travel is now ~60 Å and the route would go through solvent. Since no radical transfer can occur when β2 is in an α4β4 ring, the α4β4 state is inactive. When ATP levels rise and ATP displaces dATP in the cone domain, the ring structure breaks apart and enzymatic activity is restored.

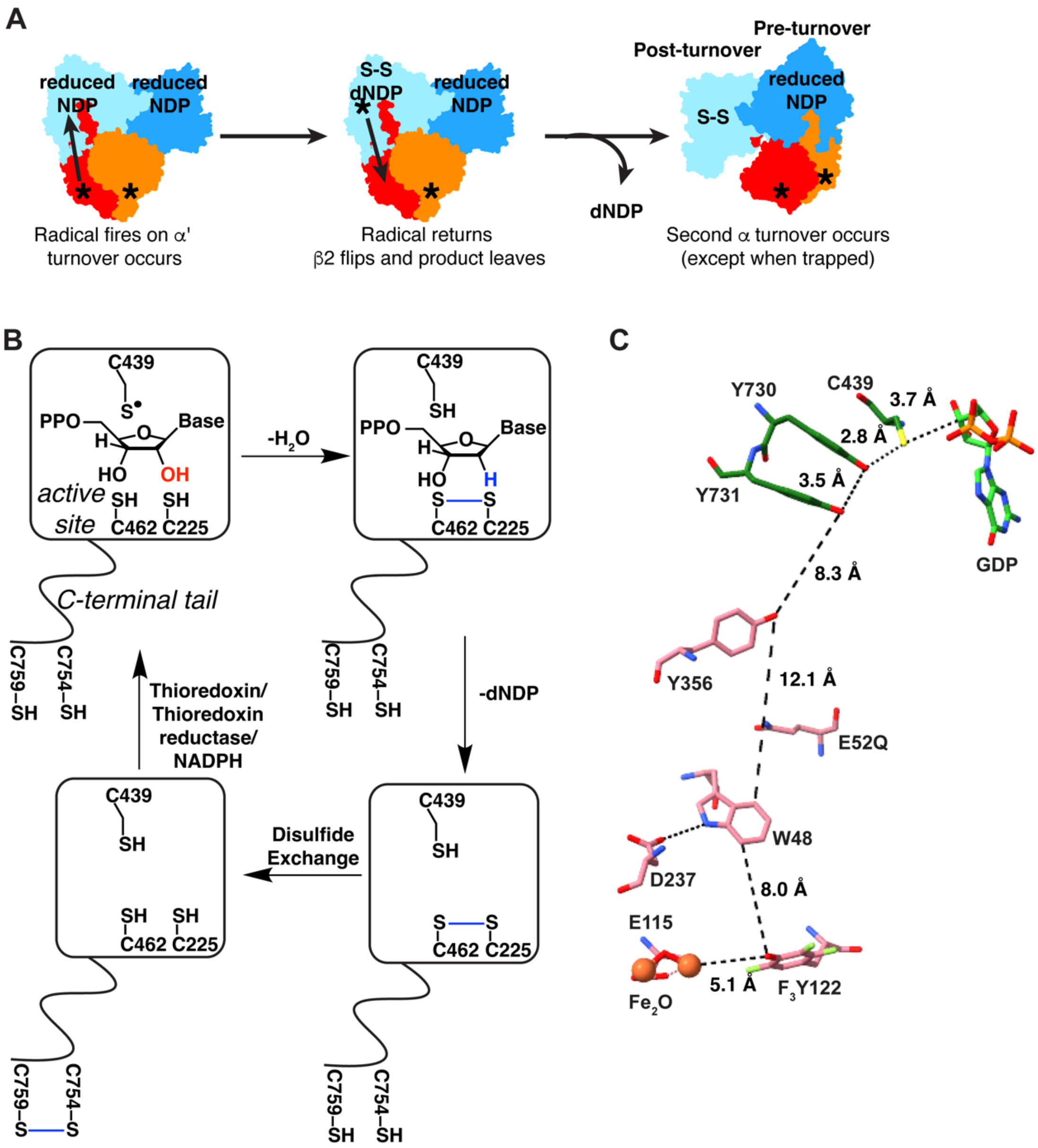

Figure S2. ***E. coli*** **class Ia RNR catalysis involves half-sites reactivity, PCET, and active site re-reduction. (A)** Schematic depiction for the half-sites reactivity of class Ia RNR that led to the previous 3.6-Å resolution cryo-EM structure. **(B)** Concomitant with nucleotide reduction in class Ia RNR, a pair of cysteines (C225, C462) in the active site are oxidized. This disulfide in the active site is re-reduced through two disulfide exchange steps, where another pair of redox-active cysteines (C754, C759) on the C-terminus of the α subunit reduces the disulfide in the active site and is subsequently re-reduced by thioredoxin/thioredoxin reductase/NADPH. **(C)** The PCET pathway of the previously published α2β2 structure at 3.6-Å resolution (PDB 6W4X) with wt-α2 and E52Q/(2,3,5)-F_3Y122-β2. The residues of the α subunit are shown with carbons in dark green and the residues of the β subunit are shown with carbons in dark pink. Interaction distances are indicated with dashed lines.

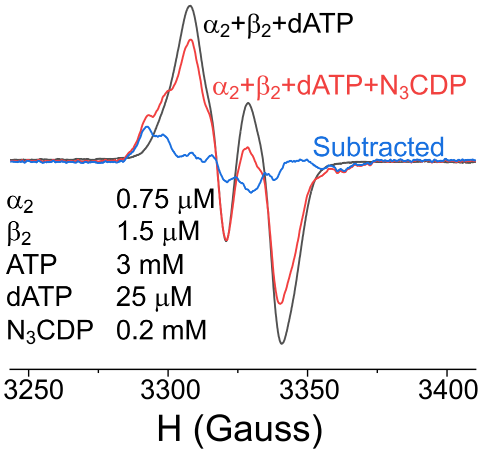

Figure S3. Electron paramagnetic resonance spectrum before and after reaction with N_3CDP. Spectrum collected using 0.75 μM α2, 1.5 μM β2, 25 μM dATP, and 3 mM ATP (black trace) and with 0.2 mM N_3CDP (red trace). The difference spectrum shown in blue was derived by subtracting the contribution of the unreacted Y122• from the composite spectrum (red trace). All samples were prepared in 50 mM HEPES pH 7.6, 15 mM $MgSO_4$, and 1 mM EDTA. In the absence of N_3CDP, the EPR signal is due to 100% Y122•. When N_3CDP is present, an N• signal contributes to the composite spectrum. Double integration of the composite (red trace) and difference (blue trace) suggests that the newly formed N• accounts for 25% of the total spin and the remaining 75% is attributed to Y122•.

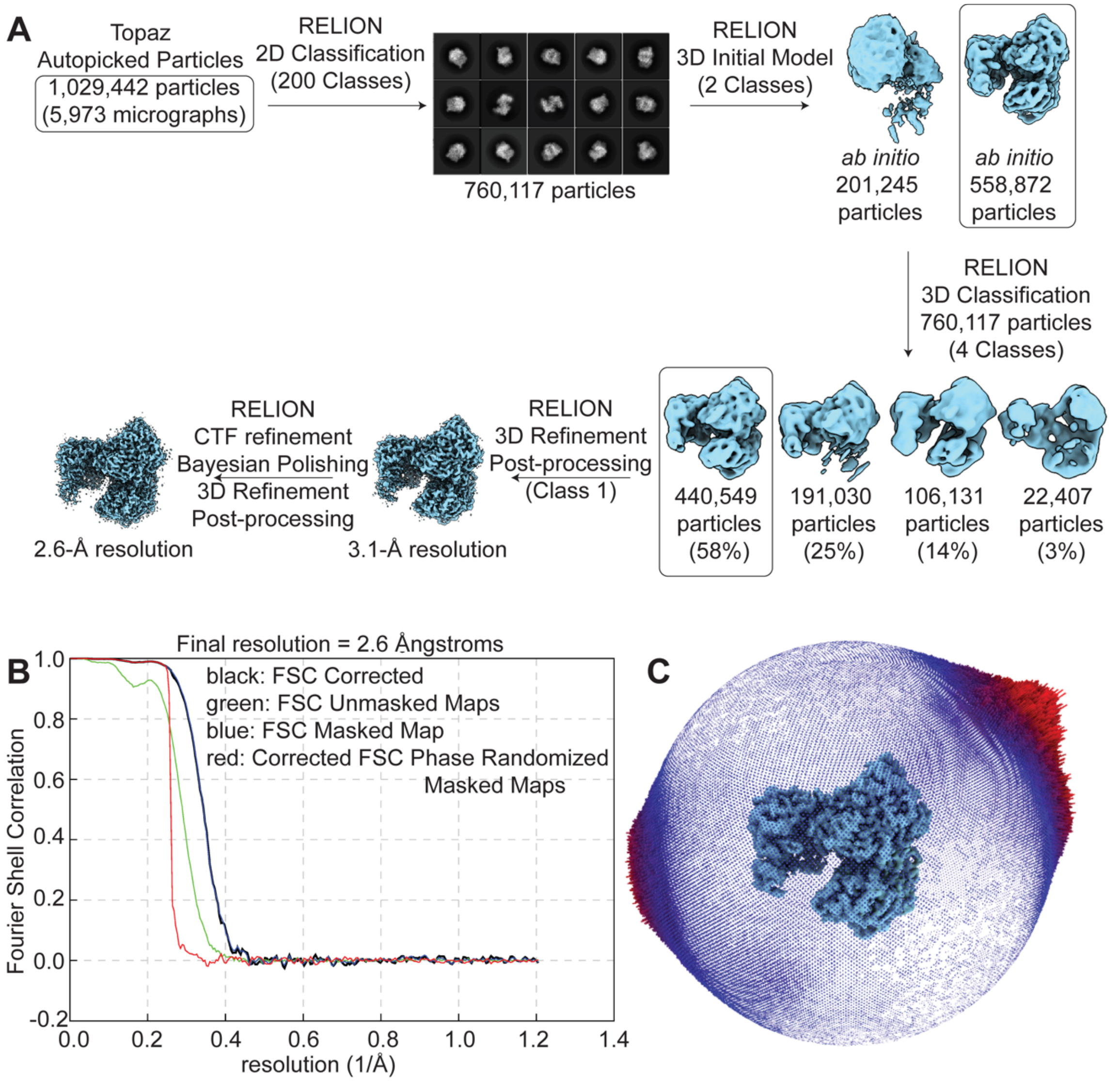

Figure S4. Cryo-EM data processing workflow. (A) Scheme describing the data processing steps to generate the final cryo-EM density map used for model building. See Methods for additional information and references to programs used. **(B)** Fourier Shell Correlation (FSC) plots for the final cryo-EM density map. **(C)** Particle orientation distribution for the final cryo-EM density map.

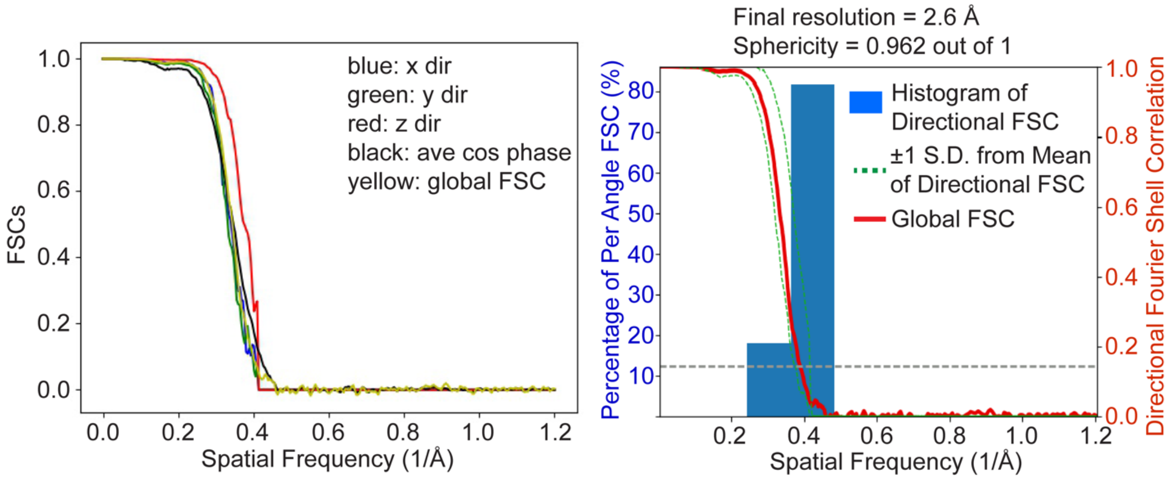

Figure S5. The 3-dimensional Fourier Shell Correlation (3DFSC) plot for the final cryo-EM 3D reconstruction shows minimal preferred orientation in the final map.

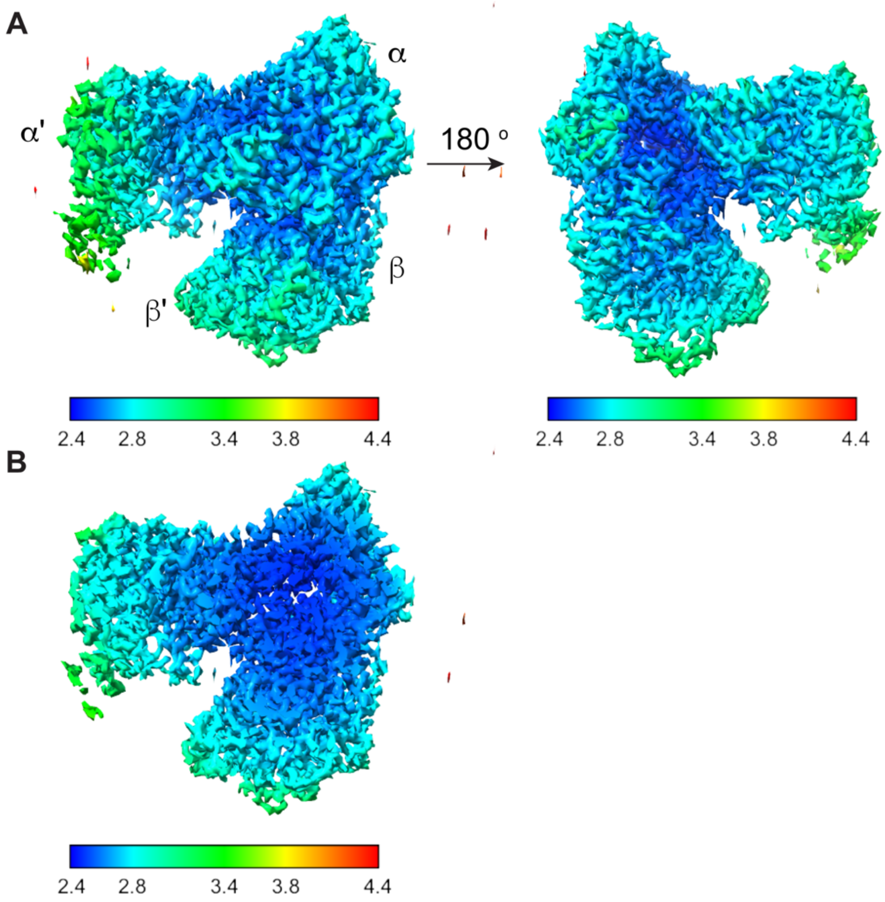

Figure S6. Local resolution maps of the final cryo-EM density map. (A) Two views, 180° rotations of each other, of the local resolution map of the final density map following sharpening by post-processing in RELION. The resolution ranges from 2.4 Å (blue) at the α2-β2 interface and core of the complex to 3.8 Å (yellow) at the N-terminus of one of the α monomers (α′ - chain A) to 4.4 Å in the solvent region. **(B)** Cross-sectional view of the final density map showing that the highest resolution (2.4 Å) is at the α2-β2 interface in the core of the complex.

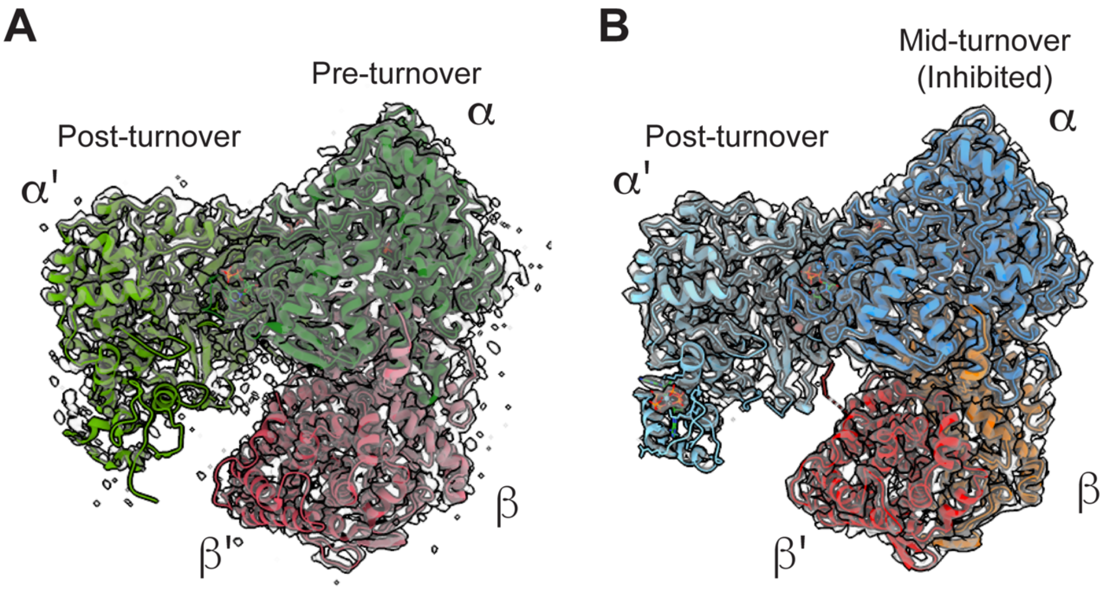

Figure S7. Comparison of *E. coli* class Ia RNR α2β2 cryo-EM structures. (A) The previously published 3.6-Å resolution cryo-EM map (transparent grey; sd level 5; PDB 6W4X) of the active state of α2 with E52Q/(2,3,5)-F_3Y122-β2 in the presence of GDP substrate and TTP specificity effector. The α2β2 model is asymmetric, where α′ (light green) and β′ (dark pink) have undergone one round of turnover and α (dark green) and β (light pink) are in a pre-turnover state which displays a fully intact radical transfer pathway. **(B)** Current structure duplicated from **Fig. 2A**.

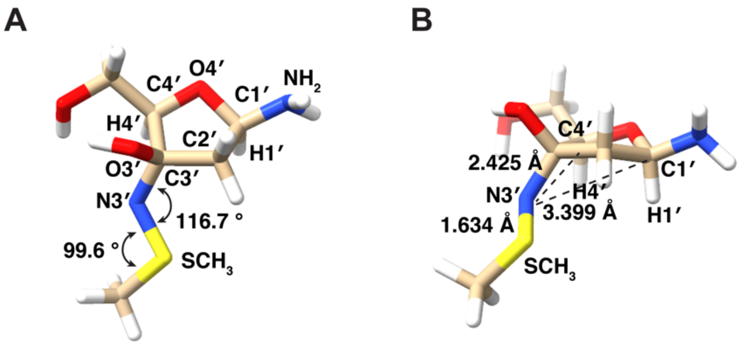

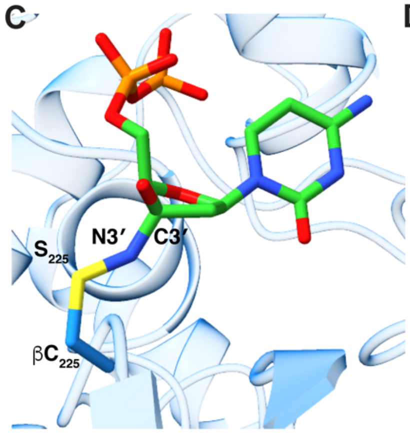

D

Distance	Spectroscopy-Based DFT Model (Å)	Refined SN-CDP (Å)
S-N3′	1.63328	1.64
C3′-N3′	1.46756	1.46
N3′-C4′	2.425	2.42
N3′-C1′	3.399	3.39

Angle	Spectroscopy-Based DFT Model (°)	Refined SN-CDP (°)
O-C3′-N3′	108.2247	108.23
C3′-N3′-S	116.6424	142.31
N3′-S-βC	99.6103	118.60

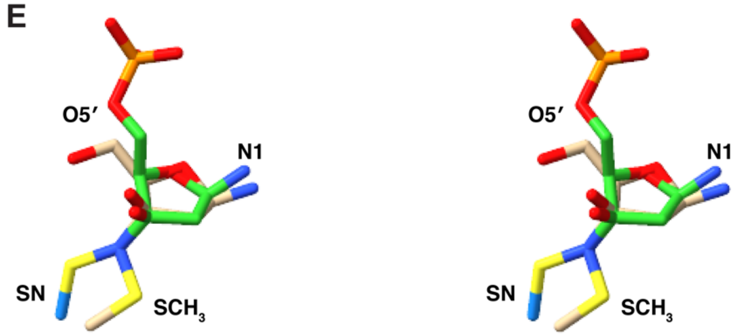

Figure S8. Comparison of spectroscopy-based density functional theory (DFT) model of adduct and cryo-EM model of SN-CDP adduct. (A) Top-down view of the DFT model of the SN-CDP intermediate. To simplify the DFT calculations, the 5′ diphosphate group of SN-CDP was approximated as a hydroxyl group, the 2′ cytidine base was approximated as a primary amine, and the cysteine was appropriately by a SCH_3 moiety (14). **(B)** Side view of the DFT model of SN-CDP. **(C)** Corresponding side view of the SN-CDP adduct following refinement into the experimental cryo-EM density. **(D)** Comparison between the distances (top) and angles (bottom) for the spectroscopy-based DFT model and the SN-CDP adduct as refined into the experimental cryo-EM density. **(E)** Stereoview of a superimposition of the spectroscopy-based DFT model (tan carbons) and the SN-CDP adduct as refined into the experimental cryo-EM density (green carbons).

A Previous structure dATP:CDP

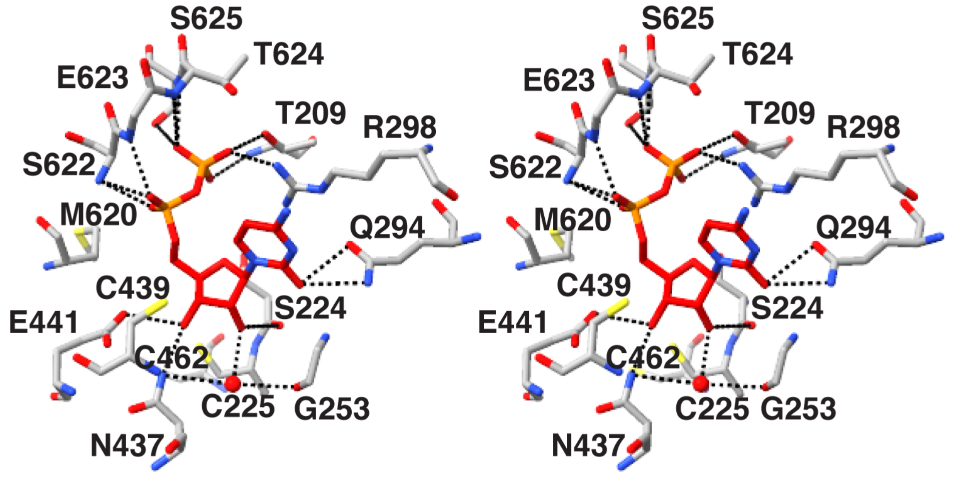

B SN-CDP adduct

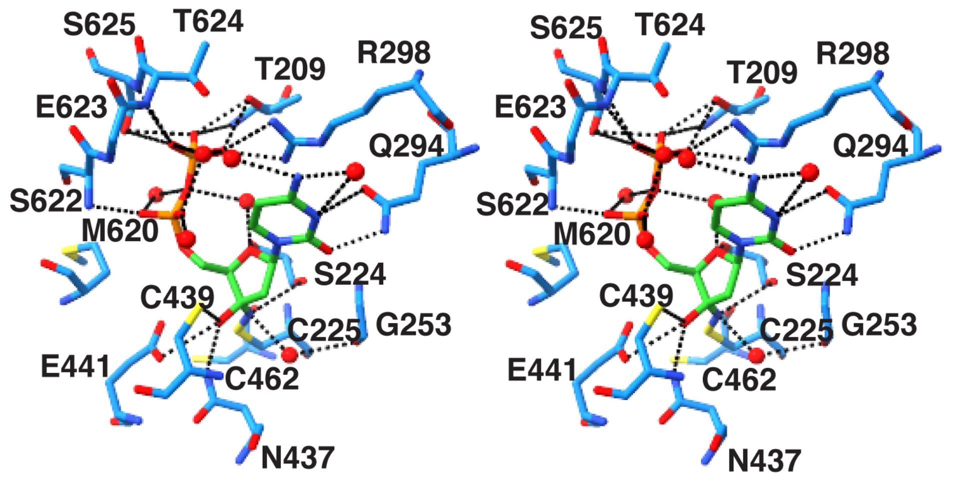

Figure S9. Stereoview comparisons of CDP binding and of SN-CDP binding. (A) Interactions between CDP substrate and the active site residues (gray carbons) from PDB 5CNS. Water molecule is shown as a red sphere. Hydrogen bonding interactions are shown with black dashed lines (distance ≤ 3.8 Å). dATP is bound in the specificity site, but is not depicted in the figure. **(B)** Interactions between the SN-CDP nucleotide (green carbons) and the active site residues (dark blue carbons) of α from this work. Water molecules are shown as red spheres. Hydrogen bonding interactions are shown with black dashed lines (distance ≤ 3.8 Å).

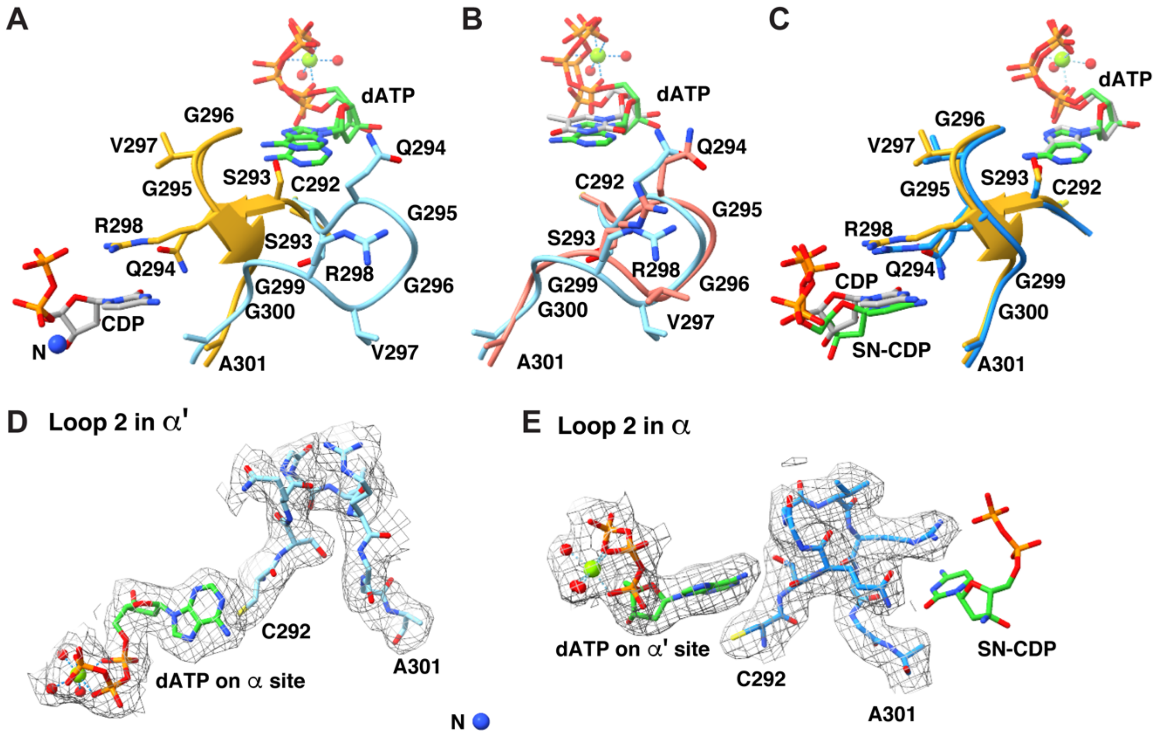

Figure S10. Loop 2 comparisons. (A) Superimposition of loop 2 in α′ (light blue, this work) and loop 2 from a previous structure with substrate-effector pair CDP-dATP bound (loop 2 in yellow, CDP with gray carbons and dATP with green carbons, PDB 5CNS). N-adduct (blue sphere) and dATP (green carbons) from current structure are also shown. **(B)** Superimposition of loop 2 in α′ (light blue, this work) and loop 2 from a previous oxidized structure (salmon, PDB 2R1R). dATP molecules are also shown with carbons in green (this work) and in gray (PDB 2R1R). **(C)** Superimposition of loop 2 in α (light blue, this work) and loop 2 from a previous structure with substrate-effector pair CDP-dATP bound (yellow, PDB 5CNS). SN-adduct and dATP (this work) are shown with carbons in green, and CDP and dATP (PDB 5CNS) are shown with carbons in gray. **(D)** Cryo-EM map (grey mesh; sd level 1) for loop 2 (light blue) of α′ and dATP bound to the adjacent specificity site. The dATP molecule is shown with carbons in green, the nitrogen atom is shown as a blue sphere, Mg^{2+} is shown as a green sphere, and water molecules are shown as red spheres. **(E)** Cryo-EM map (grey mesh; sd level 1) for loop 2 of α (dark blue) and dATP bound to the adjacent specificity site.

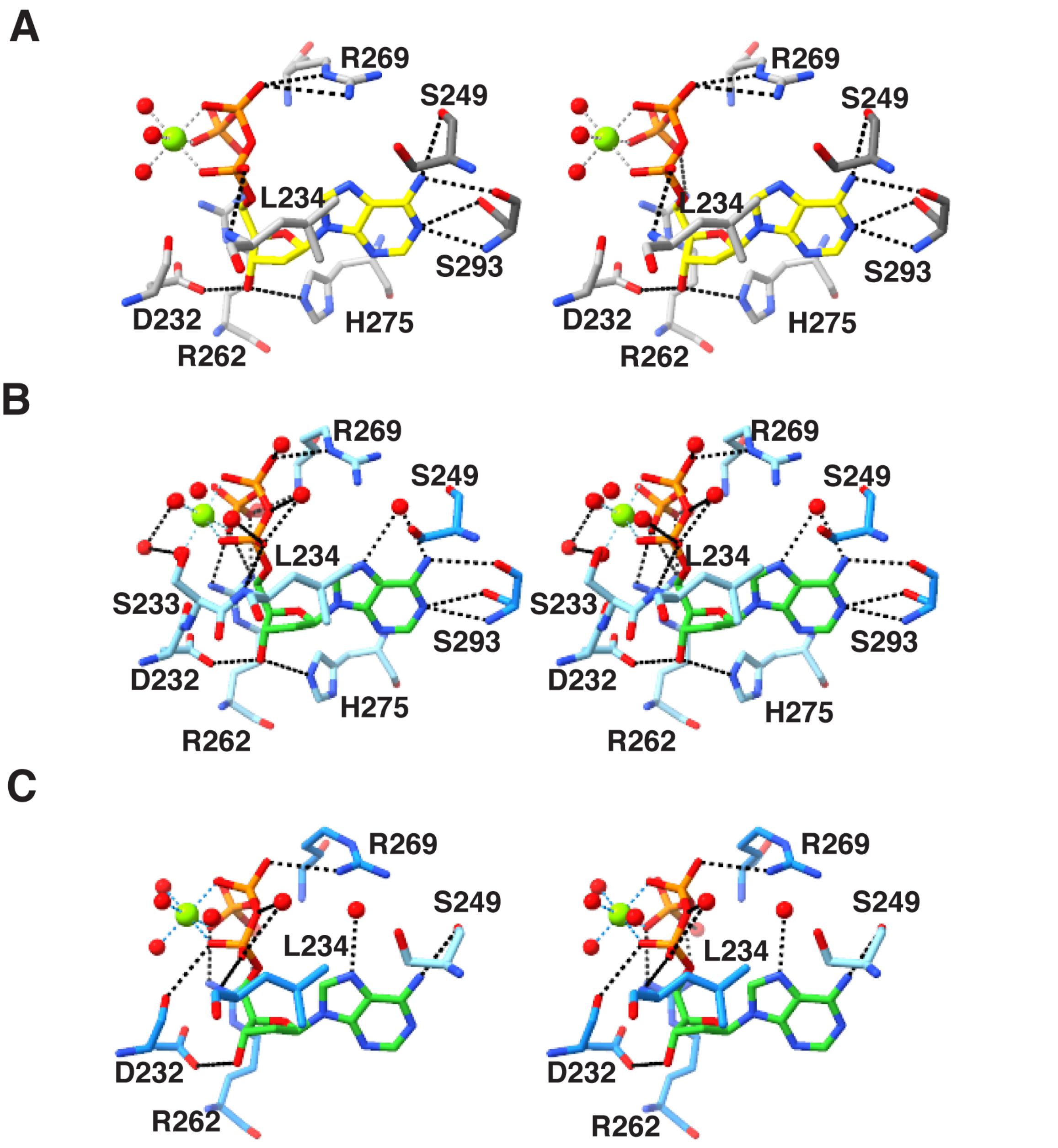

Figure S11. Stereoview comparison of structures with dATP in the specificity site. (A) dATP (yellow carbons) bound to specificity site at dimer interface from PDB 5CNS (one subunit in gray and another subunit in dark gray). Substrate CDP was bound in this structure, not shown for clarity. Water molecules are shown as red spheres and Mg^{2+} as a green sphere. Black dashed lines are possible hydrogen bonding interactions with distance ≤ 3.5 Å. **(B)** dATP (green carbons) bound in the specificity site at the dimer interface (α' in light blue, α in dark blue) that communicates with the α active site containing the SN-CDP adduct, which is not shown for clarity. Mg^{2+} is shown as a green sphere and water molecules as red spheres. Black dashed lines are possible hydrogen bonding interactions with distance ≤ 3.5 Å. **(C)** dATP (green carbons) in specificity site at dimer interface (α' in light blue, α in dark blue) that communicates with the α' active site containing the N-adduct, which is not shown for clarity. Mg^{2+} is shown as a green sphere and water molecules as red spheres. Black dashed lines are possible hydrogen bonding interactions with distance ≤ 3.5 Å.

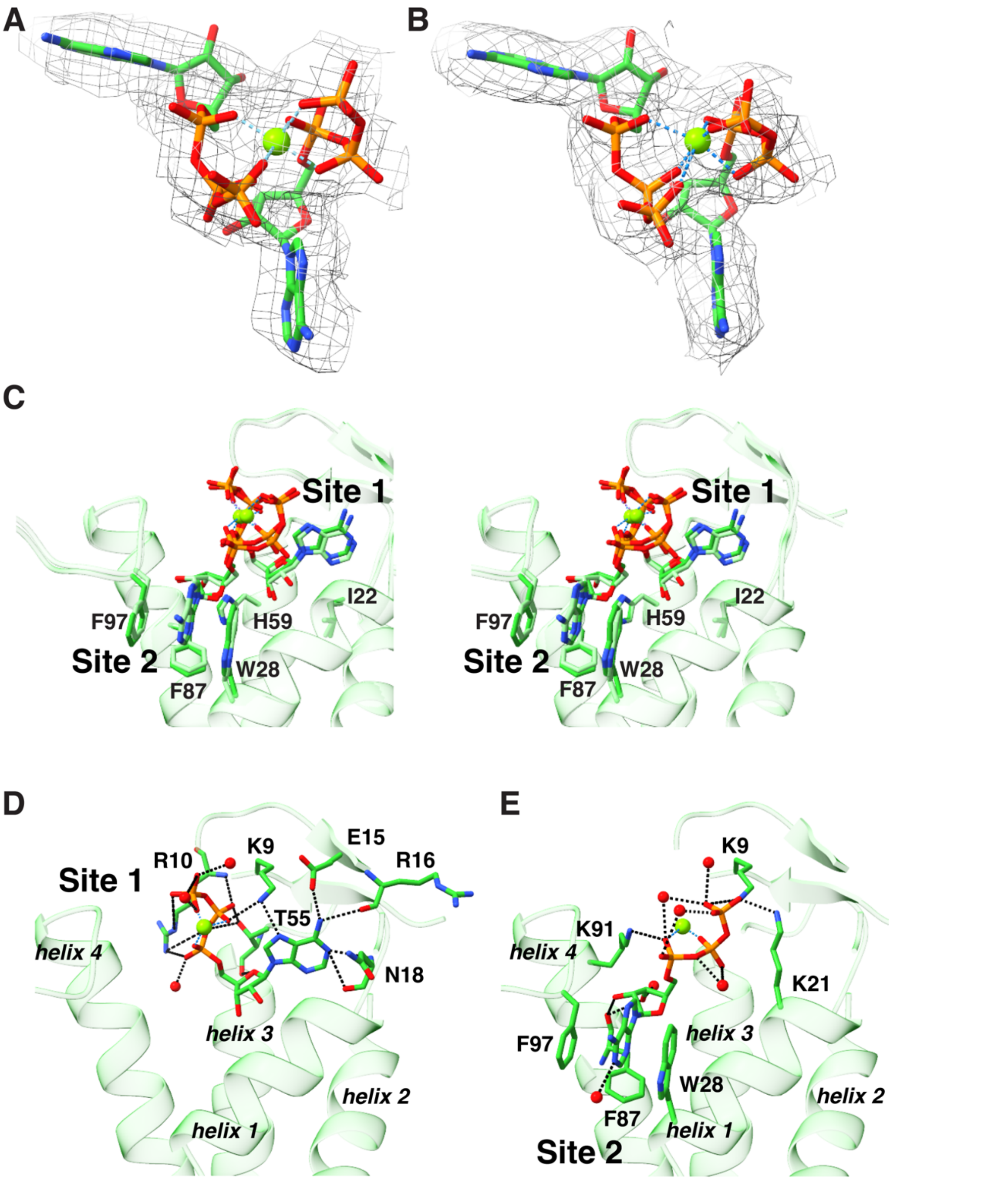

Figure S12. Two ATP molecules are bound to the activity site in the cone domain in current structure. (A) Cryo-EM map (grey mesh; sd level 1) for the two ATP molecules (green carbons) bound in the cone domain of α'. **(B)** Cryo-EM map (grey mesh; sd level 1) for the two ATP molecules (green) bound in the cone domain of α. Mg^{2+} shown as green sphere. **(C)** Stereoview of the two ATP sites in the cone domain of α (green ribbons) with interacting residues indicated. **(D)** ATP molecule (green carbons) in site 1 in the cone domain of α (green ribbons). Dashed black lines indicated potential hydrogen bonding interactions (distance ≤ 3.5 Å). The helices of the cone domain are labeled. **(E)** ATP molecule (green carbons) in site 2 in the cone domain of α (green ribbons). Note: the quality of the cryo-EM map of the cone domain is better for α than for α'.

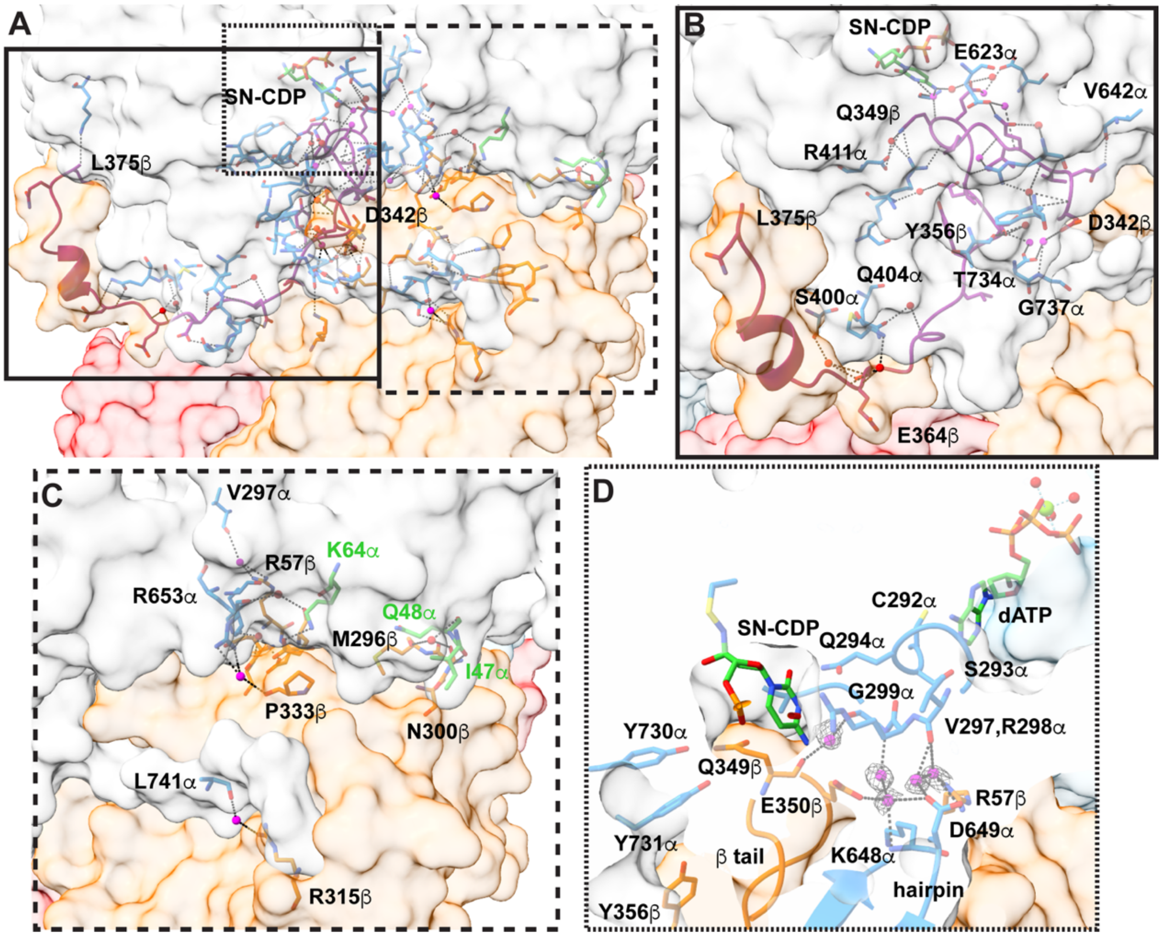

Figure S13. Bridging water molecules participate in structuring the α–β interface. (A) Surface view of the α–β interface showing amino acids participating in direct interactions and water-bridged interaction across the interface. ~3600 Å^2 of surface area on α (light gray) is buried by β (orange) with an additional ~300 Å^2 buried by β′ (red). The main interaction between β and α is made by the β-tail (residues D342 to L375). 13 of the 23 waters found at the α–β interface (**Table S11**) have been visualized previously in crystal structures of α2 or β2. Only ten water molecules are novel to the structure we describe here (shown as magenta spheres). α subunit residues are shown in dark blue, and the β subunit residues are shown in orange except for β-tail residues (D342-L375), which are shown in purple. The boxes indicate the corresponding regions shown in panels **(B**), **(C),** and **(D)**. **(B)** Zoom-in on the interfacial interactions of the β-tail with α residues either through direct interactions or through a bridging water molecule. **(C)** Zoom in on the α–β interfacial interactions that do not involve the β-tail. Labels for cone domain residues are in green. **(D)** Zoom-in on loop 2 (C292-A301) interactions with SN-CDP, specificity effector dATP (carbons in green), and water molecules (magenta spheres with gray mesh, threshold set to sd level 1). Water molecules mediate the interaction between loop 2 of α, K638 and D649 of the hairpin in α, R57 in β, and the β-tail loop (residues 342-362) that closes off the active site pocket from the aqueous environment. Hydrogen bond interactions are indicated by dashed black lines (distance ≤ 3.5 Å).

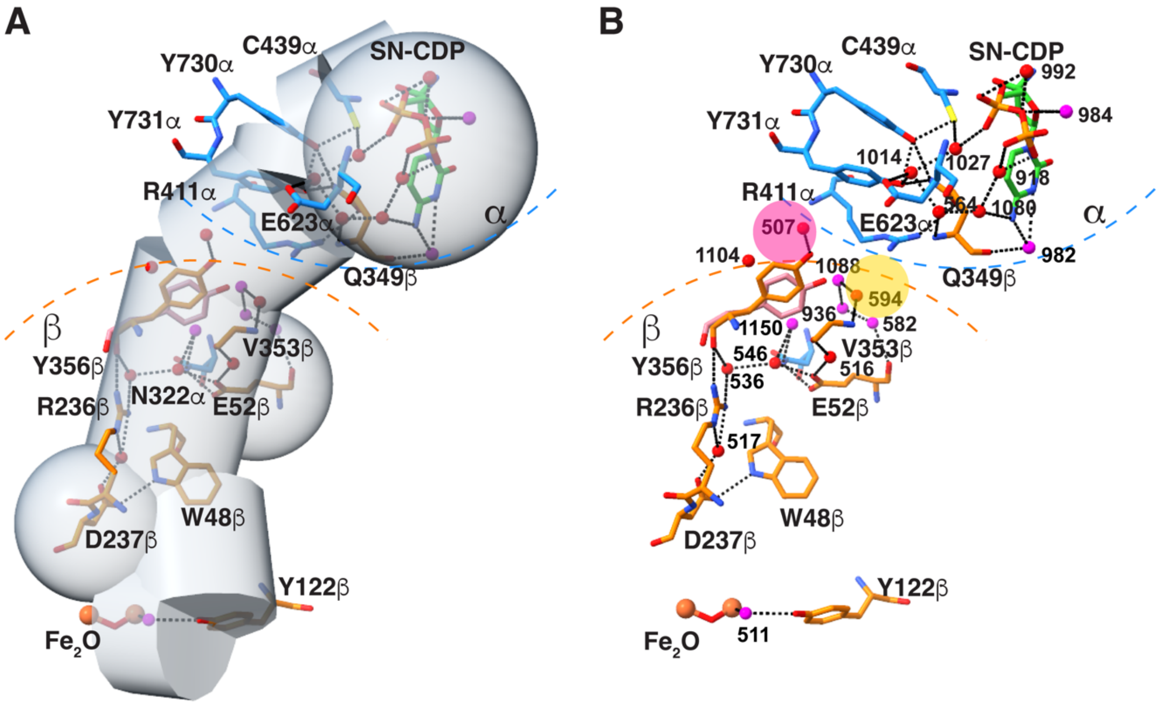

Figure S14. PCET Waters. (A) Visualization of "PCET waters," which we defined as those molecules either within a 5-Å radius of Fe_2Oβ, Y122β, D237β, W48β, E52(Q)β, Y356β, Y731α, Y730α, and C439α, and a 7-Å radius of the nucleotide or within 5 Å of the shortest path between electron transfer residue pairs. β residues are shown with orange carbons, Y356β from the previous work (PDB 6W4X) is shown with dark pink carbons, α residues are shown with dark blue carbons, and the SN-CDP adduct is shown with green carbons. The transparent gray surface encompasses the region containing PCET waters, as described in the main text. Water molecules are shown as either red spheres (previously visualized in crystal structures, **Tables S3-S6**) or magenta spheres (newly observed in this work). The black dashed lines indicate potential hydrogen bonds along the radical transfer pathway (distance ≤ 3.8 Å). **(B)** The same image as in **(A)** with water molecules labeled by number. Water molecules numbered 901-1196 correspond to chain B (α) and water molecules numbered 501-608 correspond to chain C (β). Additional hydrogen bonding interactions not depicted to preserve clarity are indicated in **Table S15**. The pink and yellow spheres indicate the direction of the start of two different water channels leading to the surface of the complex and depicted in detail in **Figure 5** and **Figure S16**.

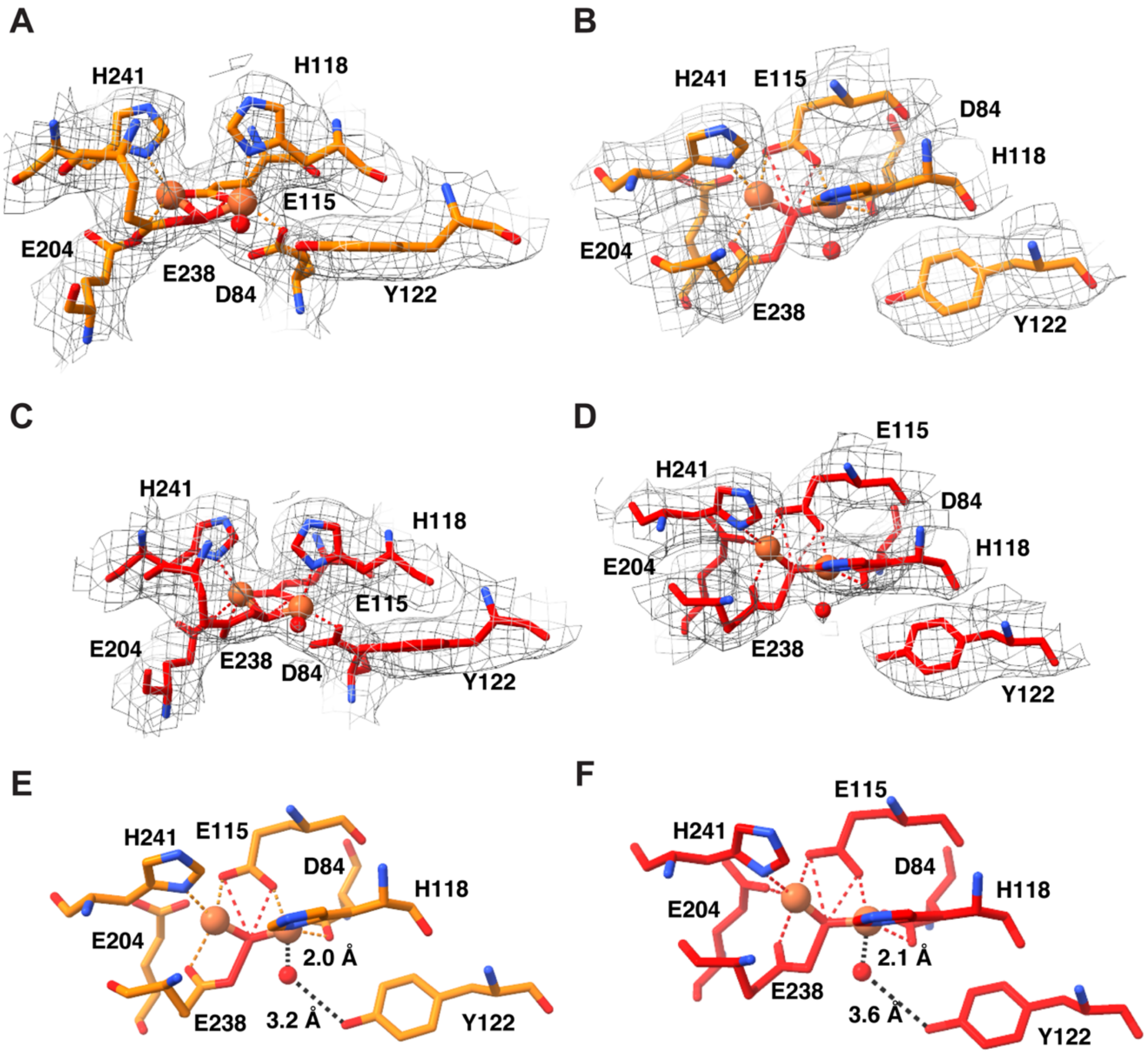

Figure S15. Cryo-EM density for the di-iron site of β2. (A) Cryo-EM map (grey mesh; sd level 1) for the di-iron site of β. The iron molecules are shown as orange spheres, the β residues are shown with orange carbons, and the water molecule is shown as a red sphere. **(B)** An orthogonal view of the di-iron site shown in **(A)**. **(C)** Cryo-EM map (grey mesh; sd level 1) for the di-iron site of β′. The iron molecules are shown as orange spheres, the β′ residues are shown with red carbons, and the water molecule is shown as a red sphere. **(D)** An orthogonal view of the di-iron site shown in **(C)**. **(E)** Interactions between the di-iron site of β (orange spheres) with the residues of β (orange carbons) and a single water molecule (red sphere). Potential hydrogen bonding interactions (distance ≤ 3.5 Å) are shown with dashed black lines and the hydrogen bonding distances for the water molecule are shown. The view is the same as in **(B)**. **(F)** Interactions between the di-iron site of β′ (orange spheres) with the residues of β′ (red carbons) and a single water molecule (red sphere). Potential hydrogen bonding interactions (distance ≤ 3.5 Å) are shown with dashed black lines and the hydrogen bonding distances for the water molecule are shown. The view is the same as in **(D)**.

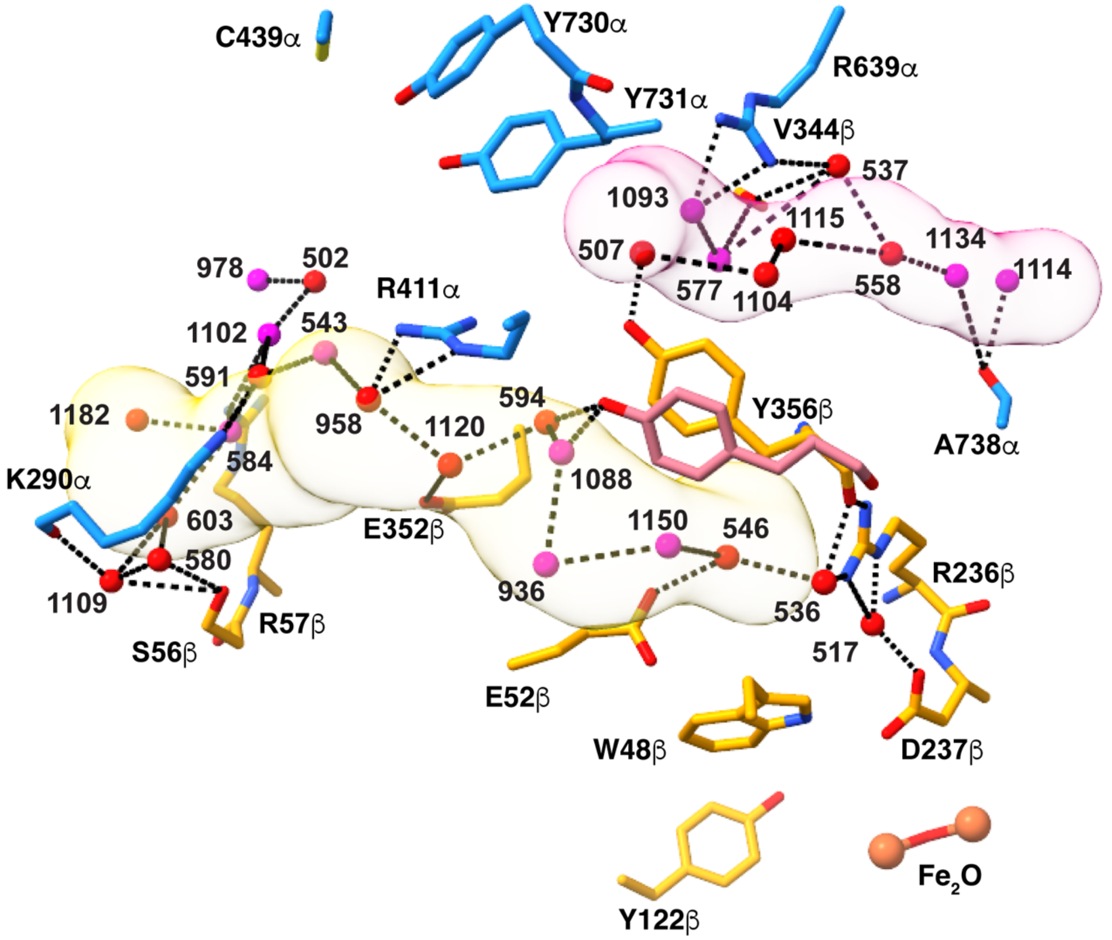

Figure S16. Water channels and networks in more detail. Figure is reproduced from **Fig. 5A** of the main text with water numbers added. The 35-Å-long yellow channel begins at Y356β, passes by E52β and E352β and exits at the back of the complex past loop 2 residue K290α. α residues are shown with carbons in dark blue and β residues are shown with carbons in orange. Previously observed water molecules are shown as red spheres and newly observed water molecules are shown as magenta spheres. The 20-Å-long pink channel runs along β-tail residues 342-356 and exits to bulk solvent between the β-tail residue D342β and G737α. Dashed black lines indicate possible hydrogen bonding interactions (distance ≤ 5 Å). Water molecules numbered 901-1196 correspond to chain B (α) and water molecules numbered 501-608 correspond to chain C (β).

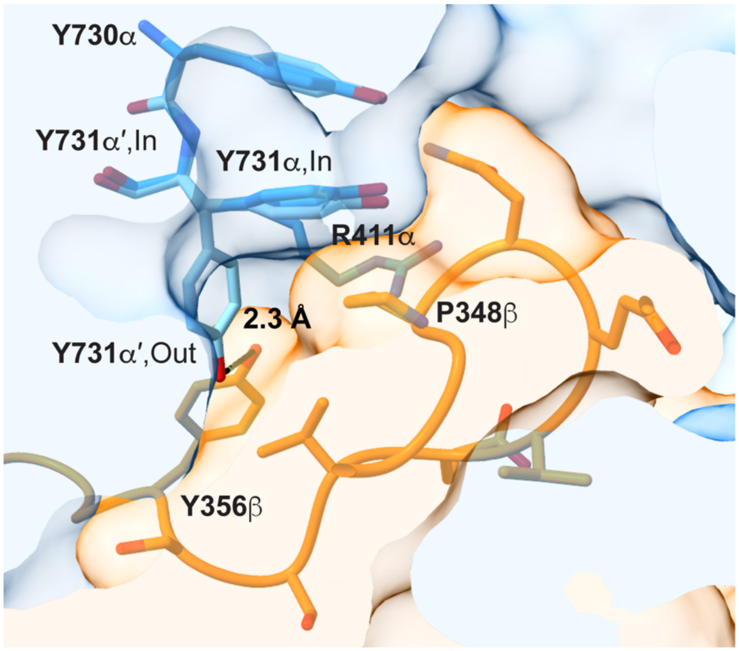

Figure S17. Surface view of α and β showing the packing of the β-tail (orange) with Y731 and Y730 of α (dark blue). Y731 and Y730 of α′ (light blue) are overlaid, including both the "flipped in" and "flipped out" conformation of Y731. The packing of P348β and R411α against Y731α appears to restrict the movement of Y731α, which is not "flipped out" when β is bound in either the previous pre-turnover or current mid-turnover structures. If Y731α "flips out" during reverse PCET, its hydroxyl group would be close to the hydroxyl group of Y356β, ~2.3-Å apart. A "flipped out" Y731α is observed post-turnover following β dissociation.

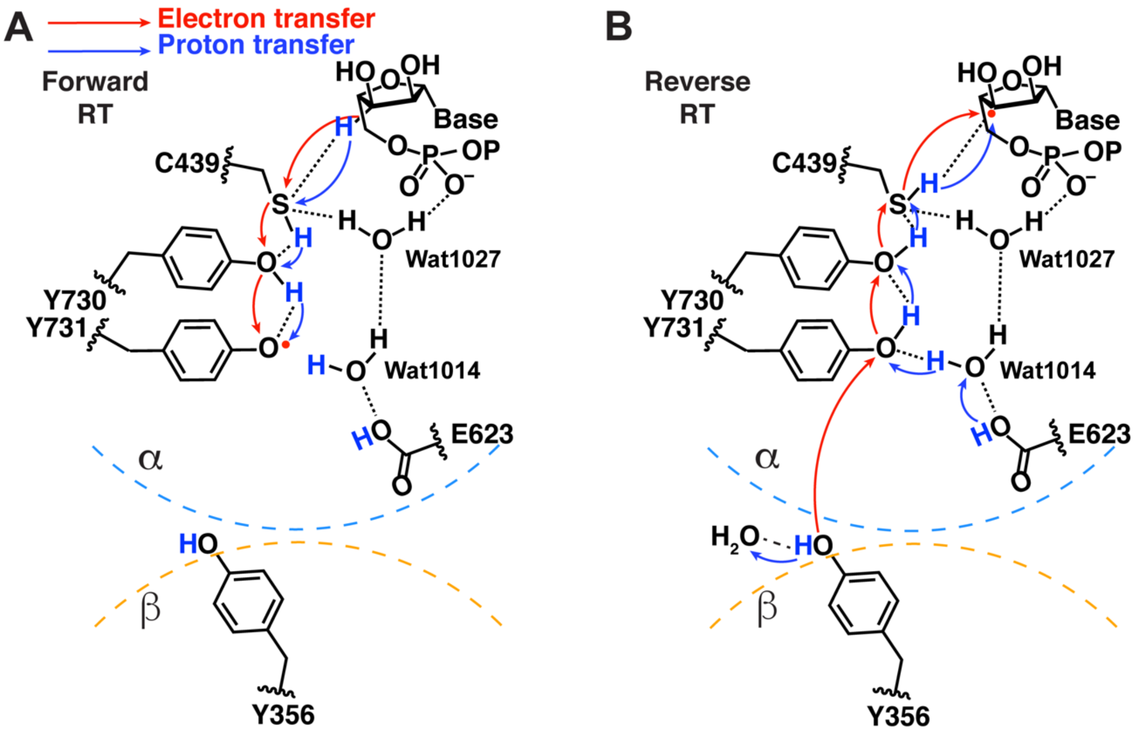

Figure S18. The water-mediated hydrogen bond network in α could facilitate colinear PCET from Y731α to the substrate. (A) Depiction of forward RT showing possible deprotonation of Y731α by E623α leading to formation of Y731α•. E623α is shown as flipped out as observed in the mid-turnover structure and water molecule Wat1014 has moved in to take its place. The presence of these water molecules (Wat1014 and Wat1027, both in chain B (α)) and this hydrogen bonding network would appear to restrict the positioning of protons of Y730α and C439α, potentially allowing for facile proton-coupled ET. **(B)** Depiction of reverse RT showing reduction of product radical to form Y356β•. The water-mediated hydrogen bond network within α appears arranged to facilitate rapid reverse radical transfer to Y356β.

Table S1. Cryo-EM data collection, reconstruction, and refinement statistics

**Accession codes**	**PDB: 9DB2; EMDB: EMD-46711; EMPIAR: EMPIAR-12249**
**Data collection**	
Microscope	FEI Titan Krios
Camera	Gatan K2
Acceleration voltage (kV)	300
Magnification (X)	105,000
Pixel size (Å)	0.415
Defocus range (μm)	-1 to -2.3
Number of frames	30
Exposure time (s)	1.2
Total exposure (e^-/Å)	50.946
Shots per hole	2
Total micrographs collected	6,007
Particles	
Micrographs used for selection	5,973
Total particles	1,029,442
**Reconstruction**	
Software	RELION-4.0
Final number of refined particles	440,549
Symmetry imposed	C1
Resolution of unmasked map (Å)	3.4
Resolution of masked map (Å)	2.6
FSC threshold	0.143
Local resolution range (Å)	2.46-4.45
**Refinement**	
Refined model resolution (Å)	2.6
dFSC model threshold	0.143
CC (mask)/CC (box)	0.90/0.66
Model composition	
Residues	2206
Water	624
SN-CDP adduct from N_3CDP	1
ATP/dATP	4/2
Fe-O-Fe	2
Mg^{2+}	4
Nitrogen resulting from N•	1
RMSD	
Bond lengths (Å)	0.005
Bond angles (°)	0.791
Molprobity score	1.74
Clashscore	11.84
Ramachandran plot (%)	
Favored	97.13
Allowed	2.73
Outliers	0.14
Rotamer Outliers (%)	0.56
CaBLAM outliers (%)	1.10
B-factors (min/max/mean)	

Protein	30.65/184.84/82.82
SN-CDP adduct from N_3CDP	64.60/83.79/73.65
ATP	78.75/197.01/136.02
dATP	57.71/74.60/66.22
Fe-O-Fe	82.93/99.18/92.58
Mg^{2+}	76.99/166.17/106.50
Nitrogen resulting from N•	59.93/59.93/59.93
Water	41.53/142.47/81.68
EMRinger Score	3.65

Table S2. The number of residues visualized in the cryo-EM structure of the RNR $\alpha2\beta2$ complex (the α subunit has 761 residues and the β subunit has 375 residues).

Subunit	Chain A (α')	Chain B (α)	Chain C (β)	Chain D (β')
α	7–739	2–745	NA	NA
β	NA	NA	1–375	1–339/360-373

Table S3. List of waters bound to chain A residues (α') that have corresponding water molecules in one or more crystal structures.

Water Number in Chain A (a')*	Location (relative to Chain A)	8VHN – Water Number in Chain A* (Chain A aligned by C-alphas)	2X0X – Water Number in Chain A (Chain A aligned by C-alphas)	1R1R – Water Number in Chain A (Chain A aligned by C-alphas)	2XAP – Water Number in Chain A (Chain A aligned by C-alphas)	2XAV – Water Number in Chain A (Chain A aligned by C-alphas)	2XAX – Water Number in Chain A (Chain A aligned by C-alphas)	2XAY – Water Number in Chain A (Chain A aligned by C-alphas)	2XAZ – Water Number in Chain A (Chain A aligned by C-alphas)	2XO4 – Water Number in Chain A (Chain A aligned by C-alphas)	2XO5 – Water Number in Chain A (Chain A aligned by C-alphas)	Qscore
902	Chain A internal	1019	2094	835	2131	2075	2061	2071	2027	2094	2101	0.785654
903	Chain A surface	N/A	2234	N/A	2320	2151	2148	2184	2069	2084	2215	0.693233
904	Chain A internal	992	2134	780	2400	N/A	N/A	N/A	N/A	2280	2142	0.826739
906	Chain A surface	1060	2073	842	2109	N/A	N/A	2060	N/A	2083	2090	0.731875
907	Chain A surface	1135	2188	N/A	2352	N/A	N/A	N/A	N/A	2246	N/A	0.868261
908	Chain A surface	N/A	N/A	N/A	N/A	N/A	2087	N/A	N/A	N/A	N/A	0.664395
909	Chain A/B interface	941	2116	N/A	2157	2080	N/A	2094	2037	2112	2121	0.882817
910	Chain A surface	N/A	N/A	N/A	N/A	N/A	2168	N/A	N/A	N/A	N/A	0.804106
911	Chain A surface	N/A	N/A	N/A	N/A	N/A	N/A	2178	N/A	N/A	N/A	0.850262
912	Chain A surface	1047	2033	N/A	2048	N/A	N/A	N/A	N/A	N/A	2051	0.72025
914	Chain A internal	N/A	2117	N/A	2159	N/A	N/A	N/A	N/A	N/A	N/A	0.792557
915	Chain A/B interface near dATP site in Chain A	1073	N/A	N/A	2148 in Chain B	N/A	N/A	N/A	N/A	N/A	N/A	0.64521
916	Chain A surface	963	2260	N/A	2362	N/A	N/A	2213	N/A	2252	2256	0.743428
917	Chain A internal	920	2108	762	2178	2098	2066	2119	2045	2139	2110	0.882335
918	Chain A surface	N/A	N/A	N/A	2265	2131	N/A	2157	N/A	N/A	N/A	0.681624
919	Chain A surface near dATP site in Chain A	1057	N/A	N/A	N/A	N/A	N/A	N/A	N/A	N/A	N/A	0.854175
920	Chain A surface	1022	2143	N/A	2195	N/A	2100	N/A	N/A	N/A	2151	0.646932
921	Chain A surface	1044	2147	N/A	2197	2101	N/A	N/A	N/A	N/A	N/A	0.75953
922	Chain A surface	1173	2020	N/A	2035	2026	2025	2027	2009	2033	2038	0.592626
923	Chain A surface	N/A	2228	N/A	2307	N/A	2004	2002	2002	2003	2005	0.785767
924	Chain A internal	1006	2257	862	2358	N/A	N/A	N/A	N/A	N/A	N/A	0.895725
925	Chain A internal	1027	2139	N/A	2189	N/A	N/A	N/A	N/A	N/A	N/A	0.778061
927	Chain A surface	959	2138	N/A	2188	N/A	N/A	N/A	N/A	N/A	2148	0.808472

928	Chain A internal	1039	2160	776	2217	2174	2111	2133	2050	2158	2167	0.856547
929	Chain A surface	1021	2162	806	2220	N/A	2110	2135	2052	2159	2168	0.670943
930	Chain A surface	N/A	2201	819	2270	2134	2133	2162	2061	2196	2193	0.707091
932	Chain A surface	1079	2294	767	2390	N/A	N/A	N/A	N/A	2249	N/A	0.828024
933	Chain A internal	999	2044	769	2079	2050	2042	2047	2018	2061	2066	0.661522
934	Chain A surface	N/A	2197	860	2266	N/A	2128	2156	N/A	2190	2189	0.806088
935	Chain A surface	N/A	N/A	N/A	N/A	N/A	2079	N/A	N/A	N/A	N/A	0.620163
936	Chain A surface	N/A	2064	N/A	2095	N/A	N/A	N/A	N/A	N/A	N/A	0.512302
937	Chain A surface	1059	2166	N/A	2224	2113	2114	N/A	2053	N/A	2171	0.845694
938	Chain A/B interface	979	2100	800	2144	2069	N/A	2077	2031	2097	2105	0.775077
939	Chain A surface	911	N/A	N/A	N/A	2110	N/A	2131	N/A	N/A	2164	0.825768
940	Chain A surface	1005	2152	N/A	2202	2102	2091	2122	N/A	N/A	N/A	0.863964
941	Chain A internal	1002	2179	771	2234	2132	2131	2159	2059	2192	2190	0.82746
942	Chain A surface	N/A	2232	N/A	2316	N/A	N/A	2181	N/A	2224	N/A	0.844911
943	Chain A internal	1037	2088	827	2127	N/A	2060	2067	2024	2090	N/A	0.787331
945	Y730/731	944	2132	845	2177	N/A	N/A	N/A	N/A	2138	N/A	0.89132
946	Chain A surface	1053	N/A	N/A	2357	N/A	N/A	N/A	N/A	N/A	N/A	0.808946
947	Chain A surface	1076	2032	N/A	2044	N/A	N/A	N/A	N/A	N/A	N/A	0.464073
948	Chain A surface	951	2161	809	2223	2112	2113	2134	2051	2163	2170	0.832905
949	Chain A surface	N/A	2059	N/A	2087	N/A	2048	2051	N/A	N/A	2082	0.841729
950	Chain A surface	1131	N/A	N/A	N/A	N/A	2032	N/A	N/A	N/A	N/A	0.563393
952	Chain A internal	1029	2296	781	2394	N/A	2180	2223	N/A	2275	2271	0.836936
953	Chain A surface	1101	2217	N/A	2292	2141	2139	N/A	N/A	2210	N/A	0.590693
955	ATP site	964	N/A	N/A	N/A	N/A	N/A	N/A	N/A	N/A	N/A	0.901812
956	Chain A internal	N/A	2126	N/A	2169	N/A	N/A	N/A	N/A	2123	N/A	0.766351
957	Chain A surface	973	2123	N/A	2165	2089	N/A	2104	N/A	2124	2133	0.887337
959	Chain A internal	1000	2170	773	2229	2116	N/A	2143	N/A	2168	2175	0.914041
960	Chain A internal	1034	2250	829	2345	N/A	N/A	2175	2068	2240	2232	0.784187
961	Chain A surface	966	2283	843	2398	N/A	N/A	N/A	N/A	N/A	2260	0.715516
962	Chain A/C interface	1043	2125	804	2167	N/A	2082	2103	2041	2126	2134	0.852138
963	dATP site in Chain C and A/B interface	1117	N/A	836	2134	N/A	N/A	2073	2029	N/A	N/A	0.857832

964	Chain A surface	1133	2236	N/A	2322	N/A	2150	N/A	N/A	2227	2266	0.857072
966	Chain A surface	955	N/A	N/A	2153	N/A	2108	N/A	N/A	N/A	N/A	0.8266
967	Chain A/C interface	1024	2129	N/A	2172	N/A	2090	2111	N/A	2127	2138	0.827116
968	Chain A internal	954	2093	772	2130	2065	N/A	2070	2026	2093	2100	0.897656
969	Chain A surface, near Chain B	N/A	N/A	N/A	2120	N/A	N/A	N/A	N/A	N/A	N/A	0.69598
970	Chain A internal	1144	N/A	N/A	N/A	N/A	N/A	N/A	N/A	N/A	N/A	0.768177
971	Chain A surface	1182	N/A	N/A	N/A	N/A	N/A	N/A	N/A	N/A	N/A	0.752754
972	Chain A/C interface	1017	2124	813	2166	N/A	2084	2105	2042	2125	N/A	0.800749
973	Chain A internal	1009	2169	777	2228	2115	N/A	2219	2082	2167	2267	0.870093
975	Chain A surface, near dATP site in Chain A	1158	N/A	N/A	N/A	N/A	N/A	N/A	N/A	N/A	N/A	0.707536
976	Chain A internal	967	2120	N/A	2160	2082	2075	2096	2038	2115	2125	0.90376
977	Chain A/B interface	N/A	2122	N/A	2162	N/A	N/A	2106 in Chain B	N/A	2142 in Chain B	2127	0.818447
978	Chain A internal	987	2300	803	2393	N/A	2183	2220	2083	2155	2268	0.850388
979	Chain A/B interface	949	2099	807	2138	2068	2064	2076	2030	2096	2104	0.937777
980	dATP site in Chain A	1192	N/A	N/A	N/A	N/A	N/A	N/A	N/A	N/A	N/A	0.63099
981	Chain A surface	1157	2243	854	2331	2145	2143	2189	N/A	2213	2222	0.840002
983	Chain A surface near ATP site	1120	2030	N/A	2041	N/A	N/A	2033	2012	2042	2044	0.382403
985	Chain A surface	N/A	2263	857	2363	N/A	N/A	N/A	N/A	N/A	N/A	0.813933
986	Chain A surface	N/A	2068	850	2100	N/A	2053	N/A	N/A	2080	N/A	0.815836
988	Chain A surface	1054	2141	N/A	2192	N/A	2097	N/A	N/A	2143	N/A	0.916273
989	Chain A/B interface	1072 in Chain B	2121 in Chain B	1195 in Chain B	2158	N/A	2059 in Chain B	2090 in Chain B	2036 in Chain B	2123 in Chain B	2122	0.852755
990	Chain A surface	N/A	2070	N/A	2308	2149	N/A	N/A	N/A	N/A	2210	0.892792
991	Chain A internal	1196	N/A	N/A	N/A	N/A	N/A	N/A	N/A	N/A	N/A	0.842984
992	Chain A internal	1004	2289	778	2387	N/A	2182	2224	N/A	2271	2265	0.894157
993	Chain A surface	N/A	N/A	N/A	2036	N/A	N/A	N/A	N/A	N/A	N/A	0.786828
994	Chain A surface	989	2103	N/A	N/A	N/A	N/A	N/A	N/A	N/A	N/A	0.837289
996	Chain A internal	1062	2231	821	2317	N/A	N/A	N/A	N/A	2223	N/A	0.857796
997	Chain A/B interface	N/A	2110 in Chain B	N/A	2146 in Chain B	N/A	N/A	N/A	N/A	N/A	N/A	0.791944

	near dATP in Chain A											
998	Chain A/B interface	1093	2098	832	2136	N/A	N/A	2074	N/A	N/A	N/A	0.823458
999	Chain A surface	N/A	N/A	N/A	N/A	N/A	N/A	N/A	N/A	N/A	2161	0.643422
1000	Chain A surface	N/A	N/A	N/A	N/A	N/A	N/A	2118	N/A	N/A	2145	0.767028
1001	Chain A surface	1095	N/A	N/A	N/A	N/A	2105	2125	N/A	2151	2158	0.715045
1002	Chain A surface	N/A	2235	N/A	2321	2152	2149	2185	2070	2226	2216	0.813629
1003	Chain A surface near dATP in Chain A	1052	N/A	N/A	N/A	N/A	N/A	N/A	N/A	N/A	N/A	0.862214
1004	Chain A/B interface	N/A	N/A	N/A	N/A	N/A	N/A	N/A	N/A	N/A	2120	0.821012
1005	Chain A/B interface	N/A	N/A	N/A	N/A	N/A	2073	N/A	N/A	N/A	N/A	0.687051
1008	Chain A surface	1068	N/A	N/A	2236	N/A	N/A	N/A	N/A	N/A	N/A	0.608874
1009	Chain A surface	N/A	2199	N/A	2268	N/A	N/A	N/A	N/A	N/A	N/A	0.822701
1010	Chain A surface	1066	N/A	N/A	N/A	N/A	N/A	N/A	N/A	N/A	N/A	0.840904
1011	Chain A surface	N/A	2103	N/A	2163	N/A	2081	2102	2040	2099	2132	0.776025
1012	Chain A surface	1137	N/A	N/A	N/A	N/A	N/A	N/A	N/A	N/A	N/A	0.484934
1013	Chain A/B interface	1074	2096	N/A	2135	N/A	N/A	N/A	N/A	N/A	N/A	0.826983
1016	Chain A surface	1138	2095	844	2132	2066	2062	2087	N/A	2095	2102	0.847849
1017	Chain A surface	N/A	2151	826	2203	N/A	N/A	N/A	N/A	2147	2153	0.772199
1018	Chain A surface	1156	2245	N/A	2333	N/A	N/A	2190	2072	2233	2223	0.690652
1020	Chain A surface	N/A	2284	N/A	2384	N/A	N/A	2216	N/A	N/A	2261	0.547331
1021	Chain A surface	1236	N/A	N/A	2276	N/A	N/A	N/A	N/A	N/A	N/A	0.712903
1022	Chain A surface	1167	2119	763	2174	2081	N/A	2113	2044	2134	2124	0.843816
1023	Chain A surface	1028	2106	N/A	N/A	2088	N/A	2083	2033	2101	N/A	0.893164
1024	Chain A surface	N/A	2240	N/A	2328	N/A	2154	2188	2071	2229	2219	0.633664
1025	Chain A surface	1110	N/A	N/A	N/A	2043	N/A	2042	N/A	N/A	N/A	0.795674
1026	Chain A surface	N/A	2253	N/A	N/A	N/A	N/A	2196	N/A	2245	N/A	0.836521
1027	Chain A surface	N/A	2154	N/A	2205	N/A	N/A	N/A	N/A	N/A	2154	0.832856
1028	Chain A/C interface	N/A	N/A	N/A	2171	N/A	N/A	2110	N/A	2132	2137	0.817144
1029	Chain A surface	N/A	2304	N/A	2408	N/A	N/A	N/A	N/A	2287	N/A	0.78381
1030	Chain A surface	1121	2255	766	2355	2160	2162	2198	N/A	2247	2241	0.810324
1031	Chain A internal	1075	2233	852	2319	N/A	2181	2183	N/A	2225	2214	0.825019

1032	Chain A surface	N/A	N/A	N/A	2084	N/A	N/A	N/A	N/A	2189	N/A	0.773776
1035	Chain A surface	N/A	2214	802	2287	2140	2138	2169	2064	2207	2200	0.919886
1036	Chain A/B interface	1123	2180	N/A	2235	N/A	N/A	N/A	N/A	N/A	N/A	0.857156
1037	Chain A surface	1116	2145	N/A	2193	2099	N/A	2120	2047	2145	2152	0.835655
1038	Chain A surface	995	2055	846	2085	N/A	N/A	2049	N/A	2066	N/A	0.892225
1039	Chain A surface	N/A	2136	N/A	2180	N/A	2092	2116	2046	2140	2143	0.854425
1041	Chain A surface	1210	2077	N/A	2116	2059	N/A	N/A	N/A	N/A	2094	0.79807
1042	Chain A internal	1144	2203	799	2272	2125	2134	2149	N/A	2179	2176	0.844352
1045	Chain A internal	1007	2076	811	2107	2058	2054	N/A	N/A	2082	N/A	0.890018
1046	Chain A surface near Chain C	905	N/A	N/A	2168	N/A	2083	N/A	N/A	N/A	N/A	0.831224
1047	Chain A surface	N/A	2146	N/A	2196	N/A	N/A	2121	2048	2146	N/A	0.886278
1049	Chain A/B interface	N/A	2039 in Chain B	N/A	2054 in Chain B	N/A	N/A	2035 in Chain B	N/A	2119	N/A	0.852794
1050	Chain A surface	N/A	2038	N/A	2057	N/A	N/A	N/A	N/A	N/A	N/A	0.789683
1008 in Chain B	Chain A/B interface	1014	2118	807	2137	N/A	2074	2075	N/A	2114	2123	0.863672
1169 in Chain B	Chain A/B interface	1138 in Chain B	N/A	N/A	2238	N/A	N/A	N/A	N/A	N/A	N/A	0.830731
526 in Chain C	Chain A/C interface	1223	N/A	N/A	N/A	N/A	N/A	N/A	N/A	N/A	N/A	0.845592
608 in Chain C	Chain A/C interface	1226	N/A	N/A	N/A	N/A	N/A	N/A	N/A	N/A	N/A	0.89588
550 in Chain D	Chain A/D interface	N/A	2158	N/A	N/A	N/A	N/A	N/A	N/A	2001	N/A	0.753131

*unless the Chain identity is otherwise specified

Table S4. List of waters bound to chain B residues (α) that have corresponding water molecules in one or more crystal structures.

Water Number in Chain B (α)*	Location (relative to Chain B)	8VHN – Water Number in Chain B (Chain B aligned by C-alphas)	2X0X – Water Number in Chain B (Chain B aligned by C-alphas)	1R1R – Water Number in Chain B (Chain B aligned by C-alphas)	2XAP – Water Number in Chain B (Chain B aligned by C-alphas)	2XAV – Water Number in Chain B (Chain B aligned by C-alphas)	2XAX – Water Number in Chain B (Chain B aligned by C-alphas)	2XAY – Water Number in Chain B (Chain B aligned by C-alphas)	2XAZ – Water Number in Chain B (Chain B aligned by C-alphas)	2XO4 – Water Number in Chain B (Chain B aligned by C-alphas)	2XO5 – Water Number in Chain B (Chain B aligned by C-alphas)	5CNS - Water Number in Chain B (Chain B aligned by C-alphas)	Qscore
901	Chain B internal	950	2197	863	2260	2060	N/A	N/A	N/A	2087	2076	N/A	0.738701
902	Chain B internal	1142	2251	N/A	N/A	N/A	N/A	N/A	N/A	N/A	N/A	N/A	0.89395
903	Chain B internal	982	2173	791	2234	2067	2049	2144	2049	2106	2085	902	0.761051
904	Chain B surface near dATP site in Chain B	1046	2150	2194	N/A	N/A	N/A	N/A	N/A	2153	2130	N/A	0.816604
906	Chain B internal	968	2114	762	2151	2068	2071	2080	N/A	2146	2086	N/A	0.839548
907	Chain B internal	1078	2208	765	2267	2151	2115	2172	N/A	2206	N/A	N/A	0.83544
908	Chain B internal	961	2256	N/A	2335	N/A	2164	N/A	N/A	2287	2243	N/A	0.812621
909	Chain B surface	1200	2155	N/A	2200	2101	2085	2124	N/A	2157	2133	N/A	0.848451
910	Chain B internal	1066	2160	N/A	2213	N/A	N/A	2131	N/A	N/A	N/A	N/A	0.787544
911	Chain B surface	1074	2048	822	2070	N/A	N/A	N/A	N/A	N/A	N/A	N/A	0.761132
912	Chain B internal	1121	2235	838	2304	N/A	N/A	N/A	N/A	N/A	2203	N/A	0.859426
913	Chain B internal	1061	N/A	N/A	N/A	N/A	N/A	N/A	N/A	N/A	N/A	N/A	0.904837
914	Chain B internal	903	2089	N/A	2115	N/A	N/A	N/A	N/A	2083	2212	N/A	0.695238
916	Chain B surface	993	2161	N/A	N/A	N/A	N/A	N/A	N/A	N/A	N/A	N/A	0.69012
917	Chain B internal	1043	2223	782	2315	N/A	N/A	2186	2071	2224	2196	904	0.623789
918	Chain B internal, in active site of Chain B	1116	N/A	N/A	2329	N/A	N/A	N/A	N/A	2248	N/A	N/A	0.491714
919	Chain B internal	959	2252	786	2336	2155	2143	2210	2083	2251	2214	N/A	0.85819
920	Chain A/B interface, near dATP site in Chain B	1025	N/A	N/A	N/A	N/A	N/A	N/A	N/A	N/A	N/A	N/A	0.659248
921	Chain B surface near Chain C	951	2261	N/A	2341	2169	N/A	2214	N/A	2255	2235	N/A	0.896796

922	Chain B internal	952	2064	770	2091	2039	2033	2046	2018	2064	2060	903	0.828868
923	Chain B internal near Chain C	931	2257	862	2334	N/A	N/A	N/A	N/A	2252	N/A	N/A	0.853353
925	Chain B surface	942	2297	789	2380	2177	2167	N/A	N/A	2293	2249	901	0.847138
927	Chain B/C interface	1088	2255	N/A	2337	N/A	2144	2211	N/A	N/A	N/A	N/A	0.87707
928	Chain B surface	1232	N/A	N/A	2061	N/A	N/A	N/A	N/A	N/A	N/A	N/A	0.783364
929	Chain B internal	924	2298	852	2372	2178	2168	2237	2095	2295	2250	N/A	0.892962
931	Chain B internal	1023	2068	846	2096	N/A	2035	N/A	N/A	N/A	N/A	N/A	0.849053
933	Chain B internal	1093	2144	780	2185	2097	2073	2113	2042	2148	2126	N/A	0.841757
934	Chain B surface	1075	N/A	N/A	2197	2099	N/A	N/A	2044	N/A	N/A	N/A	0.875564
935	Chain B surface	1103	2214	784	2277	2134	2123	2180	2065	2212	2185	N/A	0.737449
937	Chain B internal	1031	2290	803	2389	2108	N/A	N/A	N/A	2300	2242	N/A	0.802152
939	Chain B internal	1026	2301	779	2387	N/A	N/A	2241	2097	2284	2241	N/A	0.796228
940	Chain B surface	N/A	N/A	N/A	2262	2128	N/A	N/A	N/A	N/A	N/A	N/A	0.801413
941	Chain B surface	1047	2230	797	2301	N/A	N/A	N/A	N/A	2231	N/A	N/A	0.846497
942	Chain B surface	1118	N/A	N/A	N/A	N/A	N/A	N/A	N/A	N/A	N/A	N/A	0.86814
943	Chain B surface	1147	2273	N/A	2355	N/A	2154	2223	N/A	N/A	2229	N/A	0.796431
944	Chain B surface	1272	N/A	N/A	N/A	N/A	2004	2007	N/A	N/A	N/A	N/A	0.688242
945	Chain B surface	1278	N/A	N/A	N/A	N/A	N/A	N/A	N/A	N/A	N/A	N/A	0.74647
946	Chain B internal	943	2053	769	2092	2046	N/A	2053	2019	2065	2053	N/A	0.832047
947	Chain B surface	1192	N/A	N/A	N/A	N/A	N/A	N/A	N/A	N/A	N/A	N/A	0.617738
949	Chain B surface	1157	2178	N/A	2302	N/A	2127	N/A	N/A	N/A	N/A	N/A	0.582454
950	Chain B surface	N/A	2243	N/A	N/A	N/A	2135	2201	N/A	2242	N/A	N/A	0.661157
951	Chain B/C interface	N/A	2267	N/A	2079	N/A	N/A	2047	N/A	N/A	2225	N/A	0.714153
952	Chain B surface near Chain C	1003	N/A	N/A	N/A	N/A	N/A	N/A	N/A	N/A	N/A	N/A	0.73239
953	Chain B internal	902	N/A	N/A	N/A	N/A	N/A	N/A	N/A	N/A	N/A	N/A	0.854074
954	Chain B internal, in active site of Chain B	912	N/A	N/A	N/A	N/A	N/A	N/A	N/A	N/A	N/A	N/A	0.826124
957	Chain B/C interface	989	N/A	N/A	2221	N/A	N/A	N/A	N/A	N/A	N/A	N/A	0.853547
958	Chain B/C interface	1099	N/A	N/A	N/A	2093	N/A	N/A	N/A	N/A	N/A	N/A	0.848245

959	Chain B internal	919	N/A	N/A	N/A	N/A	N/A	N/A	N/A	N/A	N/A	N/A	0.853978
960	Chain B internal	953	2205	829	2324	N/A	N/A	2171	N/A	2205	2180	N/A	0.848027
961	Chain B/C interface	N/A	2095	N/A	2128	N/A	N/A	N/A	N/A	N/A	N/A	N/A	0.792468
962	Chain B surface	967	2271	851	2352	N/A	N/A	N/A	N/A	N/A	N/A	N/A	0.832754
964	Chain B surface	1055	2220	783	2286	2139	2125	2185	N/A	2223	2195	N/A	0.870325
966	Chain B surface	1133	N/A	N/A	N/A	N/A	N/A	N/A	N/A	N/A	N/A	N/A	0.777318
967	Chain B internal	972	2287	778	2373	2180	N/A	2239	2096	2298	2240	N/A	0.844779
968	ATP site	937	N/A	N/A	N/A	N/A	N/A	N/A	N/A	N/A	N/A	N/A	0.908857
969	Chain B/C interface	1040	2262	857	2342	N/A	2146	2215	N/A	2256	2228	N/A	0.863627
970	ATP site	935	N/A	N/A	N/A	N/A	N/A	N/A	N/A	N/A	N/A	914	0.527132
971	Chain B internal	1038	2209	N/A	2268	N/A	N/A	N/A	2062	2207	2181	N/A	0.713339
972	Chain B surface	1110	N/A	N/A	N/A	N/A	N/A	N/A	N/A	N/A	N/A	N/A	0.643336
973	Chain B internal	921	N/A	N/A	2160	N/A	N/A	N/A	N/A	N/A	N/A	N/A	0.915434
974	Chain B surface	1015	2168	831	2225	2112	2094	N/A	N/A	2176	N/A	N/A	0.919044
975	Chain B surface	1246	2008	N/A	2010	N/A	N/A	2010	N/A	N/A	2014	N/A	0.688661
976	Chain B surface	N/A	2281	N/A	N/A	N/A	N/A	N/A	N/A	N/A	N/A	N/A	0.802981
980	Chain B surface near ATP site	1206	2037	N/A	2053	N/A	N/A	N/A	N/A	N/A	2047	N/A	0.803467
983	Chain B surface	N/A	2148	N/A	2215	N/A	2078	N/A	N/A	2151	N/A	N/A	0.716411
985	Chain B internal	975	2180	771	2237	2115	2102	2152	2054	2186	2159	906	0.783604
986	Chain B surface	1194	2015	N/A	2017	2013	2010	2016	2005	2019	2019	N/A	0.518359
987	Chain B surface	962	2204	819	2266	2131	2114	2170	N/A	2204	2179	N/A	0.837626
988	Chain A/B interface	998 in Chain A	2118	N/A	2159	2078	2058	2088	N/A	2121	2102	N/A	0.813145
989	Chain B surface near chain D	907	2164	N/A	2216	N/A	2088	2135	N/A	2167	2143	N/A	0.823397
991	Chain B internal	1073	2177	773	2233	N/A	N/A	2147	2052	2181	2155	907	0.882765
992	Chain B internal, in active site of Chain B	N/A	N/A	793	2007	N/A	N/A	N/A	N/A	N/A	N/A	N/A	0.823473
993	Chain B surface	920	2195	N/A	2257	N/A	N/A	N/A	N/A	N/A	N/A	N/A	0.814889
995	Chain B internal	1137	2293	801	2377	N/A	N/A	N/A	N/A	2289	2245	N/A	0.879423
996	Chain A/B interface	1010	N/A	N/A	N/A	N/A	N/A	N/A	N/A	N/A	N/A	N/A	0.830866

998	Chain B internal	998	2292	781	2376	N/A	N/A	N/A	N/A	2288	2244	N/A	0.866307
999	Chain A/B interface near dATP site in Chain B	992	N/A	836	2140	2070	2048	2078	2032	2117	2096	N/A	0.749727
1000	Chain B surface	947	2295	N/A	2363	2176	2158	2226	N/A	2290	2236	N/A	0.731393
1001	Chain B internal	1009	2285	812	2367	2171	2163	2229	2092	2279	2239	N/A	0.851709
1002	Chain B internal	1062	2288	775	2385	2174	N/A	2240	N/A	2283	2150	N/A	0.789614
1003	Chain B surface	1020	2250	N/A	2331	N/A	N/A	N/A	2082	N/A	N/A	N/A	0.81577
1004	Chain B surface near Chain C	N/A	2024	N/A	N/A	N/A	2015	2022	N/A	2024	2030	N/A	0.887079
1005	Chain B surface	956	N/A	N/A	2318	N/A	N/A	N/A	N/A	N/A	N/A	N/A	0.789051
1006	Chain B internal	1146	N/A	N/A	2117	N/A	N/A	N/A	N/A	N/A	N/A	N/A	0.865334
1007	Chain B internal	1079	2184	772	2246	N/A	N/A	2155	2056	2190	2079	N/A	0.911185
1009	Chain B surface	N/A	2203	N/A	2265	2129	2112	2168	2060	2202	2178	N/A	0.658598
1010	Chain B internal	1065	2176	777	2232	N/A	2098	2146	2051	2180	2154	N/A	0.840747
1011	Chain B surface	1115	2210	N/A	2272	N/A	N/A	N/A	N/A	N/A	N/A	N/A	0.926666
1012	ATP site	908	N/A	N/A	N/A	N/A	2001	N/A	N/A	N/A	N/A	N/A	0.60108
1013	Chain B surface	N/A	2069	N/A	2099	N/A	N/A	N/A	N/A	2071	N/A	N/A	0.530241
1014	Chain B/C interface, in active site of Chain B	1170	N/A	N/A	N/A	N/A	N/A	N/A	N/A	N/A	N/A	N/A	0.814412
1015	Chain A/B interface	N/A	2119	N/A	N/A	N/A	N/A	N/A	N/A	N/A	2103	N/A	0.834008
1017	Chain B internal	1053	2275	849	2359	N/A	2157	N/A	2062	2276	2233	N/A	0.681621
1019	Chain B/C interface	N/A	2259	792	2340	2156	N/A	2213	2085	2254	2217	N/A	0.76307
1020	Chain B surface	N/A	N/A	N/A	2089	N/A	N/A	N/A	N/A	N/A	2043	N/A	0.834909
1021	Chain B surface	N/A	N/A	N/A	N/A	N/A	N/A	N/A	N/A	2031	N/A	922	0.705861
1022	Chain B surface near Chain D	N/A	N/A	N/A	N/A	2091	N/A	N/A	N/A	2143	2121	N/A	0.572681
1023	Chain B surface	N/A	N/A	N/A	N/A	N/A	N/A	N/A	N/A	2034	2057	N/A	0.808067
1024	Chain B surface	941	2131	N/A	2172	2083	2065	2100	N/A	2134	2113	N/A	0.857824
1025	Chain B internal, in active site of Chain B	1076	2174	N/A	2150	2113	2097	2145	2050	2104	2152	905	0.911641
1026	Chain B internal	1081	2269	768	2349	2042	2151	2220	2021	2267	2227	N/A	0.880544

1027	Chain B/C interface, in active site of Chain B	N/A	N/A	N/A	N/A	N/A	N/A	N/A	N/A	N/A	N/A	923	0.812897
1028	Chain B internal	1105	2254	767	2333	N/A	N/A	N/A	N/A	N/A	2215	N/A	0.860179
1029	Chain B/C interface	1195	2258	N/A	2338	N/A	2145	N/A	2084	2253	N/A	N/A	0.808222
1030	Chain B surface	N/A	N/A	N/A	2106	N/A	N/A	N/A	2078	N/A	N/A	N/A	0.62857
1031	Chain B surface	N/A	N/A	N/A	2174	N/A	N/A	N/A	N/A	N/A	N/A	N/A	0.823983
1032	Chain B/C/D interface	N/A	N/A	N/A	N/A	N/A	2090	N/A	N/A	N/A	N/A	N/A	0.867879
1033	Chain B surface	N/A	2082	N/A	2039	N/A	N/A	N/A	2026	2079	2070	N/A	0.825628
1034	Chain B surface	1131	N/A	N/A	N/A	N/A	N/A	N/A	N/A	2198	N/A	N/A	0.868278
1035	Chain B surface	1158	2056	820	2077	2041	N/A	2209	2020	2059	2056	N/A	0.880316
1036	Chain B surface	1259	2200	860	2263	N/A	N/A	N/A	N/A	N/A	2176	N/A	0.810512
1039	Chain B surface	N/A	2084	818	2109	N/A	N/A	2064	2027	N/A	2072	N/A	0.792416
1040	Chain B/C interface	1234	2123	N/A	2163	2094	2070	2011	2037	2145	N/A	N/A	0.821964
1041	Chain B surface	1017	2282	843	2368	N/A	N/A	N/A	N/A	2280	N/A	N/A	0.779527
1043	Chain B internal	1128	2299	798	2384	2179	2169	2238	N/A	2296	N/A	N/A	0.893644
1044	Chain B internal, near active site in Chain B	N/A	2187	N/A	2251	2120	N/A	2158	2058	2194	2166	909	0.866504
1045	Chain B surface	1036	2244	854	2319	N/A	N/A	2202	N/A	2243	2208	N/A	0.599221
1046	Chain B/C interface	N/A	2147	N/A	2189	2098	2077	2117	N/A	2150	2128	N/A	0.893947
1047	Chain B surface	964	2083	850	2108	2050	N/A	N/A	N/A	N/A	N/A	N/A	0.874061
1049	Chain B surface	1175	N/A	N/A	2149	2065	2110	N/A	N/A	N/A	N/A	N/A	0.826325
1050	Chain B internal	1002	2300	776	2386	2110	2092	N/A	2048	2297	2148	913	0.865613
1051	Chain B surface	N/A	2163	N/A	N/A	2107	N/A	2132	N/A	2164	N/A	N/A	0.79693
1053	ATP site	1139	N/A	N/A	N/A	N/A	N/A	N/A	N/A	N/A	N/A	N/A	0.683631
1054	Chain B surface	N/A	2159	826	2206	2103	N/A	2126	N/A	2159	2136	N/A	0.873995
1056	Chain B/C interface	N/A	N/A	N/A	N/A	N/A	N/A	2207	2080	2250	N/A	N/A	0.841839
1058	Chain B surface	N/A	N/A	N/A	2059	N/A	2021	N/A	N/A	N/A	N/A	N/A	0.907003
1059	Chain B surface	1243	2202	N/A	2264	N/A	N/A	N/A	N/A	N/A	N/A	N/A	0.768359
1061	Chain B surface	1124	2169	806	2223	2109	2093	2143	N/A	2177	2149	N/A	0.802367
1062	Chain B surface	1172	2154	N/A	2201	N/A	2084	2123	2045	2156	2134	N/A	0.779686
1063	Chain B internal	1071	2227	N/A	2292	2125	2126	2189	N/A	2227	2199	925	0.860836

1064	Chain B surface	1257	2219	N/A	2285	2137	N/A	2184	2069	2219	2193	N/A	0.935318
1065	Chain B surface	1213	N/A	N/A	N/A	N/A	N/A	N/A	N/A	2050	N/A	N/A	0.723453
1066	Chain B surface	990	2156	N/A	2203	2102	N/A	2125	N/A	2158	2135	N/A	0.698498
1067	Chain B internal	981	2185	839	2244	N/A	2099	N/A	2057	2189	2156	N/A	0.786205
1068	Chain B internal	N/A	2207	799	2247	2118	N/A	N/A	N/A	2191	2164	N/A	0.859488
1072	Chain B surface	N/A	2233	N/A	N/A	N/A	N/A	N/A	N/A	N/A	N/A	N/A	0.906241
1074	Chain B internal	1173	2245	766	2325	N/A	N/A	2212	N/A	2246	N/A	N/A	0.855388
1075	Chain B internal	944	2238	821	2309	N/A	N/A	N/A	N/A	2235	N/A	N/A	0.785574
1077	Chain B surface	N/A	2188	N/A	2252	N/A	N/A	N/A	N/A	N/A	N/A	N/A	0.871595
1078	Chain B internal	1014	2086	764	2112	2054	N/A	2065	N/A	2081	2073	N/A	0.905924
1079	Chain B surface	N/A	2032	N/A	2045	N/A	N/A	N/A	N/A	2032	2037	N/A	0.770573
1080	Chain B/C interface, in active site of Chain B	N/A	2247	N/A	N/A	N/A	N/A	N/A	N/A	N/A	N/A	N/A	0.836946
1082	Chain A/B interface	1107	2132	804	2173	2084	N/A	2101	N/A	2135	2112	N/A	0.763866
1085	Chain B surface	1114	2153	N/A	2198	N/A	N/A	N/A	N/A	2052	2132	N/A	0.764913
1089	Chain B surface	N/A	2215	833	2279	N/A	2119	N/A	N/A	2216	2186	N/A	0.763415
1091	Chain A/B interface	1186	N/A	N/A	N/A	N/A	N/A	N/A	N/A	N/A	N/A	N/A	0.839061
1092	Chain B surface, near Chains A and C	1190	2139	763	2182	2092	N/A	N/A	N/A	N/A	2123	924	0.942153
1094	Chain B internal	1210	2067	788	N/A	N/A	N/A	N/A	N/A	N/A	N/A	N/A	0.741976
1095	Chain B surface	1186	2152	N/A	2199	N/A	2083	2122	N/A	N/A	2131	N/A	0.853415
1096	Chain B surface	1179	2165	N/A	2222	N/A	N/A	2141	N/A	2173	2147	N/A	0.783163
1097	Chain B/C interface	1199	2263	N/A	N/A	N/A	N/A	N/A	N/A	2257	N/A	911	0.915354
1098	Chain B surface	1082	N/A	N/A	2258	N/A	2108	N/A	N/A	N/A	N/A	N/A	0.797896
1099	Chain B internal	1060	N/A	N/A	N/A	N/A	N/A	N/A	N/A	N/A	N/A	N/A	0.866521
1100	Chain B surface near Chain A	1039	2138	N/A	2181	2090	N/A	N/A	N/A	N/A	N/A	N/A	0.865435
1101	ATP site	1229	2035	N/A	2052	N/A	N/A	2034	2016	2039	2040	N/A	0.807395
1104	Chain B/C interface, near Y356 in Chain C	N/A	2304	N/A	2391	N/A	N/A	N/A	N/A	N/A	N/A	N/A	0.738173
1105	Chain A/B interface	N/A	2092	N/A	2120	2059	N/A	2071	N/A	2085	N/A	N/A	0.882834

1106	Chain B surface near Chain C	1205	2240	N/A	2311	2145	2132	2198	2074	2239	2205	N/A	0.600165
1107	Chain B surface	1167	N/A	N/A	2068	N/A	N/A	N/A	N/A	N/A	N/A	N/A	0.86576
1108	Chain B surface	1102	2041	N/A	2056	N/A	N/A	N/A	N/A	N/A	N/A	N/A	0.823467
1109	Chain A/B/C interface	1051	2124	N/A	2162	N/A	N/A	N/A	N/A	N/A	2105	N/A	0.891692
1110	Chain B/C/D interface	N/A	N/A	N/A	N/A	N/A	N/A	N/A	N/A	2169	N/A	N/A	0.807915
1111	Chain B surface near Chain C	1252	2031	N/A	2006	2025	2017	2029	2014	2030	2035	N/A	0.85353
1112	Chain B surface	901	2055	N/A	2076	2040	2031	N/A	N/A	N/A	2055	N/A	0.85184
1113	Chain B internal	1070	2085	811	2111	N/A	N/A	N/A	N/A	2080	N/A	N/A	0.877702
1115	Chain B/C interface	N/A	N/A	N/A	N/A	2171	N/A	N/A	N/A	N/A	N/A	N/A	0.807249
1116	Chain B surface	1117	2054	842	2075	N/A	N/A	N/A	N/A	2058	N/A	N/A	0.920671
1117	Chain B/C interface	N/A	2028	N/A	2038	2024	N/A	2028	N/A	N/A	N/A	N/A	0.883843
1118	Chain B surface near dATP site in Chain B	1123	2104	844	2137	N/A	N/A	N/A	N/A	2097	2081	N/A	0.823314
1120	Chain B/C interface	N/A	2136	N/A	2177	N/A	N/A	2102	N/A	2137	2115	N/A	0.794066
1121	Chain B internal	1301	N/A	N/A	N/A	N/A	N/A	N/A	N/A	N/A	N/A	N/A	0.869759
1122	Chain B surface	1100	N/A	N/A	N/A	N/A	N/A	N/A	N/A	N/A	N/A	N/A	0.905213
1123	Chain B/C interface	N/A	2098	840	2344	2158	2018	2217	2086	2092	2036	N/A	0.627721
1125	Chain B surface	N/A	N/A	N/A	N/A	N/A	N/A	N/A	N/A	2166	2142	N/A	0.90189
1126	Chain B surface	N/A	N/A	N/A	2057	N/A	N/A	N/A	N/A	N/A	N/A	N/A	0.82522
1127	Chain B surface	960	2241	N/A	2313	N/A	2133	2199	2075	2240	2206	N/A	0.815075
1128	ATP site	1165	N/A	N/A	N/A	N/A	N/A	N/A	N/A	N/A	2092	N/A	0.827876
1129	dATP site in Chain B	1187	N/A	N/A	N/A	N/A	N/A	N/A	N/A	N/A	N/A	N/A	0.800928
1130	Chain B internal	1160	N/A	N/A	N/A	N/A	N/A	N/A	N/A	N/A	N/A	N/A	0.800537
1131	Chain B surface	1129	N/A	N/A	2051	2028	2019	N/A	N/A	N/A	N/A	N/A	0.739785
1133	Chain B surface	1135	2229	N/A	2295	2126	2107	N/A	N/A	2197	2173	N/A	0.913668
1135	Chain B internal	948	N/A	N/A	N/A	N/A	N/A	N/A	N/A	N/A	N/A	N/A	0.799553
1136	Chain B/C interface	1183	2289	N/A	2388	N/A	N/A	N/A	N/A	2285	N/A	N/A	0.870009
1137	Chain B/C interface	N/A	2019	N/A	2024	N/A	2012	N/A	N/A	N/A	N/A	N/A	0.886737
1138	Chain A/B interface	1087	2106	N/A	2140	2064	N/A	2078	2029	2100	N/A	N/A	0.900034

1139	Chain B/C interface	N/A	N/A	N/A	N/A	N/A	N/A	N/A	N/A	2028	N/A	N/A	0.849285
1140	Chain B/C interface	973	N/A	N/A	N/A	N/A	2066	N/A	N/A	N/A	N/A	N/A	0.745589
1142	Chain A/B interface near dATP site in Chain A	1141	2111	N/A	2148	N/A	N/A	N/A	N/A	N/A	2083	N/A	0.754389
1143	Chain B internal	980	2239	N/A	2310	N/A	N/A	N/A	N/A	2237	N/A	N/A	0.89543
1144	Chain A/B interface and dATP site in Chain B	1241	2110	N/A	N/A	N/A	N/A	2079	N/A	2116	N/A	N/A	0.846162
1145	Chain B surface	N/A	2158	N/A	2208	N/A	N/A	2128	N/A	2160	2125	N/A	0.8945
1146	Chain B surface	1089	2224	N/A	2289	N/A	N/A	2187	N/A	2225	2197	N/A	0.801791
1148	Chain B/C interface	N/A	2025	N/A	2034	N/A	N/A	N/A	N/A	2026	N/A	N/A	0.90239
1149	Chain B internal	N/A	N/A	N/A	2093	N/A	N/A	N/A	N/A	2066	2074	N/A	0.832765
1152	Chain B surface	1045	2284	N/A	2369	2172	2162	2230	2093	2281	2238	N/A	0.750837
1153	Chain B surface	1270	2107	N/A	2141	N/A	N/A	N/A	N/A	2101	N/A	N/A	0.850887
1157	Chain B/C interface	N/A	2268	N/A	2036	2023	2152	2219	2089	2266	2224	N/A	0.89614
1158	Chain B surface	1239	N/A	N/A	2202	N/A	N/A	N/A	N/A	N/A	N/A	N/A	0.902143
1159	Chain B surface near chain C	N/A	2296	N/A	2378	N/A	N/A	N/A	N/A	2291	N/A	N/A	0.900754
1160	Chain B surface	N/A	N/A	N/A	2269	N/A	N/A	N/A	N/A	N/A	N/A	N/A	0.800748
1161	Chain A/B interface	1207	2108	832	2143	N/A	N/A	N/A	N/A	N/A	N/A	921	0.853925
1163	Chain B surface	1282	N/A	N/A	N/A	N/A	N/A	N/A	N/A	N/A	N/A	N/A	0.798455
1166	Chain B surface near Chain C	N/A	N/A	N/A	N/A	N/A	N/A	N/A	N/A	N/A	2256	N/A	0.896872
1167	Chain B surface	1143	N/A	N/A	2245	N/A	N/A	N/A	N/A	N/A	N/A	N/A	0.674744
1170	Chain A/B interface	1111	2181	800	2238	N/A	N/A	2151	2055	2185	N/A	N/A	0.773511
1171	Chain B surface	1244	N/A	N/A	N/A	N/A	N/A	N/A	N/A	N/A	N/A	N/A	0.819553
1172	Chain B surface	N/A	2072	815	2287	2138	N/A	N/A	2070	2209	2065	N/A	0.91076
1173	Chain B surface	1306	N/A	N/A	2175	N/A	N/A	N/A	N/A	N/A	2111	N/A	0.812918
1175	Chain B/C interface	N/A	2096	N/A	2129	N/A	N/A	N/A	N/A	N/A	N/A	N/A	0.73625
1176	Chain B internal	1231	2170	814	2228	N/A	N/A	N/A	N/A	N/A	N/A	N/A	0.722855
1179	Chain B internal in active site of Chain B	1269	2090	N/A	N/A	N/A	N/A	N/A	N/A	N/A	N/A	N/A	0.890412

1180	Chain B surface	1230	N/A	N/A	N/A	N/A	N/A	N/A	N/A	N/A	N/A	N/A	0.679166
1181	Chain B/D interface	N/A	N/A	N/A	N/A	N/A	N/A	2108	N/A	N/A	N/A	N/A	0.787115
1182	Chain A/B/C interface	1228	N/A	N/A	N/A	N/A	N/A	N/A	N/A	N/A	N/A	N/A	0.720529
1183	Chain B surface	1317	N/A	790	N/A	2133	2039	2177	2063	N/A	N/A	N/A	0.742122
1184	Chain B surface	N/A	N/A	N/A	N/A	2123	N/A	N/A	N/A	N/A	N/A	N/A	0.635074
1185	Chain B surface	1309	N/A	N/A	N/A	N/A	2117	N/A	N/A	N/A	N/A	N/A	0.870323
1188	Chain B surface	1083	2228	N/A	2294	2142	N/A	2191	2072	N/A	2200	N/A	0.741399
1189	Chain B surface	N/A	N/A	N/A	N/A	2124	N/A	2162	N/A	N/A	2172	N/A	0.741347
1192	Chain B surface	1314	N/A	N/A	N/A	N/A	N/A	N/A	N/A	N/A	N/A	N/A	0.940014
1193	Chain B surface	1244	N/A	N/A	N/A	N/A	N/A	N/A	N/A	N/A	N/A	N/A	0.832208
1194	Chain B surface	1298	N/A	N/A	N/A	N/A	N/A	N/A	N/A	N/A	N/A	N/A	0.939378
1195	Chain A/B interface near Chain C	1313	2120	N/A	2042	N/A	N/A	2036	N/A	N/A	2046	N/A	0.812362
1196	Chain B surface	1292	N/A	N/A	N/A	N/A	N/A	N/A	N/A	N/A	N/A	N/A	0.842742
901 in Chain A	Chain A/B interface	N/A	N/A	N/A	N/A	2076	N/A	N/A	N/A	N/A	N/A	N/A	0.814448
974 in Chain A	Chain A/B interface near dATP site in Chain B	1186	N/A	N/A	N/A	N/A	N/A	N/A	N/A	N/A	N/A	N/A	0.815534
982 in Chain A	Chain A/B interface	1042	2109	N/A	2139	N/A	N/A	N/A	N/A	2102	N/A	N/A	0.792635
1006 in Chain A	Chain A/B/C interface	N/A	N/A	N/A	N/A	N/A	N/A	N/A	N/A	N/A	N/A	912	0.873525
1007 in Chain A	Chain A near dATP site in Chain A	1091	N/A	N/A	N/A	N/A	N/A	N/A	N/A	N/A	N/A	N/A	0.874661
1043 in Chain A	Chain A/B interface	N/A	2012	N/A	N/A	N/A	N/A	N/A	N/A	N/A	N/A	N/A	0.735162
1048 in Chain A	Chain A surface near dATP site in Chain A	N/A	N/A	N/A	N/A	N/A	N/A	N/A	N/A	N/A	2114 in Chain A	N/A	0.759753
503 in Chain C	Chain B/C interface	1261	2130	N/A	2171	N/A	N/A	N/A	N/A	N/A	N/A	N/A	0.851865
506 in Chain C	Chain B/C interface	1164	2001 in Chain E	N/A	2002 in Chain E	N/A	N/A	2234	N/A	2001	2002 in Chain E	N/A	0.813433
507 in Chain C	Chain B/C interface near Y356 in Chain C	N/A	2303	N/A	N/A	2182	2170	2140	N/A	2302	2252	N/A	0.815922
514 in Chain C	Chain B/C interface	N/A	N/A	N/A	2005 in Chain E	N/A	N/A	N/A	N/A	N/A	N/A	N/A	0.69363
537 in Chain C	Chain B/C interface	1161	2277	N/A	2362	N/A	2159	2227	2091	2277	N/A	N/A	0.745016

544 in Chain C	Chain B/C interface	N/A	N/A	N/A	2029	N/A	N/A	N/A	N/A	N/A	2023	N/A	0.855975
553 in Chain C	Chain A/B/C interface	N/A	N/A	N/A	2058	N/A	N/A	N/A	N/A	N/A	N/A	N/A	0.775084
558 in Chain C	Chain B/C interface	N/A	N/A	N/A	2393	N/A	N/A	2244	N/A	2305	2234	N/A	0.730446
564 in Chain C	Chain B/C interface in the active site of Chain B	N/A	N/A	N/A	N/A	N/A	N/A	N/A	N/A	2299	N/A	N/A	0.794003
573 in Chain C	Chain B/C/D interface	N/A	N/A	N/A	2218	N/A	N/A	2137	N/A	2171	N/A	N/A	0.873128
580 in Chain C	Chain A/B/C interface	1030	N/A	N/A	N/A	N/A	N/A	N/A	N/A	N/A	N/A	N/A	0.731432
591 in Chain C	Chain B/C interface in active site of Chain B	1305	N/A	N/A	N/A	N/A	N/A	2098	N/A	2131	N/A	N/A	0.915874
594 in Chain C	Chain B/C interface near Y356 in Chain B	N/A	N/A	N/A	N/A	N/A	N/A	2139	N/A	2172	2146	N/A	0.896096
603 in Chain C	Chain A/B/C interface	N/A	2127	816	N/A	N/A	2061	2094	N/A	2127	N/A	N/A	0.811685
604 in Chain C	Chain B/C interface	N/A	N/A	N/A	N/A	2163	N/A	N/A	N/A	N/A	N/A	N/A	0.904331

*unless the Chain identity is otherwise specified

Table S5. List of waters bound to chain C residues (β) that have corresponding water molecules in one or more crystal structures.

Water Number in Chain C (β)*	Location (relative to Chain C)	2XOF – Water Number in Chain A (Chain A aligned by C-alphas)	5CI0 – Water Number in Chain A (Chain A aligned by C-alphas)	5CI1 – Water Number in Chain A (Chain A aligned by C-alphas)	5CI2 – Water Number in Chain A (Chain A aligned by C-alphas)	5CI3 – Water Number in Chain A (Chain A aligned by C-alphas)	5CI4 – Water Number in Chain A (Chain A aligned by C-alphas)	5CNS – Water Number in Chain H (Chain H aligned by C-alphas)	5CNS – Water Number in Chain G (Chain G aligned by C-alphas)	Qscore
501	Chain C surface	2155	635	621	640	646	669	N/A	N/A	0.69721
504	Chain C surface	2052	N/A	N/A	N/A	N/A	N/A	N/A	N/A	0.748096
505	Chain C internal	N/A	533	509	516	508	506	N/A	N/A	0.827597
508	Chain C internal	2119	562	517	532	553	527	608	607	0.843476
510	Chain B/C interface	2189	N/A	N/A	N/A	N/A	N/A	N/A	N/A	0.882875
512	Chain C internal	2072	511	548	525	569	516	N/A	N/A	0.812895
515	Chain C internal	2013	588	645	595	586	577	N/A	610	0.8421
516	Chain B/C interface	N/A	678	505	581	679	710	N/A	N/A	0.827894
517	Chain C internal	2135	535	545	539	552	558	609	604	0.743141
518	Chain C surface	N/A	N/A	669	N/A	N/A	633	N/A	N/A	0.885654
522	Chain C internal	2132	605	549	592	604	603	603	N/A	0.907498
523	Chain C surface	N/A	671	698	N/A	N/A	680	N/A	N/A	0.864723
524	Chain C surface	N/A	670	676	650	579	667	N/A	N/A	0.761002
525	Chain C surface	2177	617	510	537	551	637	N/A	N/A	0.789202
527	Chain C surface	2063	568	531	582	581	624	N/A	N/A	0.790612
528	Chain C surface near Chain A	N/A	N/A	N/A	N/A	512	502	N/A	N/A	0.799775
529	Chain B/C interface	2163	508	558	509	583	574	N/A	N/A	0.82611
530	Chain C internal	2137	586	597	605	601	610	N/A	612	0.894852
531	Chain C/D interface	2078	513	546	571	572	524	N/A	N/A	0.879885
532	Chain C surface	2058	536	N/A	562	518	553	N/A	N/A	0.725901
533	Chain C/D interface	N/A	679	530	558	532	568	N/A	N/A	0.838667
534	Chain C surface	2178	650	639	600	574	530	N/A	N/A	0.567009
536	Chain B/C interface	N/A	570	584	594	635	534	N/A	N/A	0.840254
538	Chain C surface	2151	645	659	644	642	617	N/A	N/A	0.74493
541	Chain C internal	2116	623	623	626	585	627	N/A	N/A	0.803239

545	Chain B/C interface	2162	614	577	646	641	620	N/A	N/A	0.863681
546	Chain B/C interface	N/A	689	666	688	684	608	N/A	N/A	0.84668
547	Chain C internal	2023	607	652	658	628	609	N/A	613	0.889909
548	Chain C/D interface	N/A	555	628	623	503	645	N/A	N/A	0.903091
551	Chain B/C interface	2133	N/A	N/A	N/A	N/A	N/A	N/A	N/A	0.846489
552	Chain C surface near Chain B	2032	578	604	611	576	660	N/A	N/A	0.90908
554	Chain C surface	2031	675	660	659	634	643	N/A	N/A	0.559846
555	Chain C surface	2165	616	646	616	645	592	N/A	N/A	0.88504
557	Chain C/D interface	2081	629	N/A	N/A	589	N/A	N/A	N/A	0.886191
559	Chain C surface	2067	661	624	548	677	677	N/A	N/A	0.806189
560	Chain C surface	2051	N/A	N/A	N/A	N/A	N/A	N/A	N/A	0.845962
566	Chain C internal	2143	549	611	565	538	541	N/A	N/A	0.753506
568	Chain C/D interface	N/A	630	626	641	682	666	N/A	N/A	0.813004
569	Chain C internal	2117	564	593	535	570	536	N/A	N/A	0.848924
571	Chain C/B interface	2014	677	690	687	698	676	N/A	N/A	0.861487
574	Chain C surface	2138	636	544	609	575	539	N/A	N/A	0.681999
575	Chain B/C interface	2018	565	529	577	632	551	N/A	N/A	0.887211
576	Chain B/C interface	N/A	632	550	612	588	569	N/A	N/A	0.84851
579	Chain C surface	2101	503	603	512	511	511	N/A	N/A	0.869885
581	Chain C surface	2171	639	671	645	622	646	N/A	N/A	0.851588
583	Chain B/C interface	2175	600	600	578	547	586	N/A	N/A	0.782821
585	Chain C/D interface	N/A	596	585	637	623	616	N/A	N/A	0.817923
587	Chain C/D interface	N/A	633	683	675	671	638	N/A	N/A	0.866949
588	Chain C internal	2149	527	552	544	599	543	N/A	N/A	0.809273
590	Chain C surface	2110	631	658	663	656	651	N/A	N/A	0.835024
593	Chain C surface	2026	667	633	649	666	682	N/A	N/A	0.789744
595	Chain C/D interface	2083	581	504	575	540	533	N/A	N/A	0.903847
598	Chain C internal	2144	N/A	N/A	N/A	N/A	N/A	N/A	N/A	0.792783

601	Chain C internal	2036	643	650	629	560	566	N/A	N/A	0.891445
1028 in Chain A	Chain A/C interface	2046	609	632	614	611	N/A	N/A	N/A	0.817144
1048 in Chain B	Chain B/C interface	2042	N/A	N/A	N/A	N/A	N/A	N/A	N/A	0.921024
1057 in Chain B	Chain B/C interface	N/A	697	701	700	706	703	N/A	N/A	0.868964
1073 in Chain B	Chain B/C interface	N/A	693	689	694	694	700	N/A	N/A	0.77985
506 in Chain D	Chain C/D interface	2093	N/A	N/A	N/A	N/A	N/A	N/A	N/A	0.793798
525 in Chain D	Chain C/D interface	2079	N/A	620	632	N/A	658	N/A	N/A	0.869497
527 in Chain D	Chain C/D interface	2104	N/A	572	536	521	N/A	N/A	N/A	0.907282
537 in Chain D	Chain C/D interface	N/A	602	516	608	607	601	N/A	N/A	0.924188
559 in Chain D	Chain C/D interface	2043	N/A	N/A	N/A	N/A	N/A	N/A	N/A	0.905344

*unless the Chain identity is otherwise specified

Table S6. List of waters bound to chain D residues (β') that have corresponding water molecules in one or more crystal structures.

Water Number in Chain D (β')*	Location (relative to Chain D)	2XOF – Water Number in Chain B (Chain B aligned by C-alphas)	5CI0 – Water Number in Chain A (Chain A aligned by C-alphas)	5CI1 – Water Number in Chain A (Chain A aligned by C-alphas)	5CI2 – Water Number in Chain A (Chain A aligned by C-alphas)	5CI3 – Water Number in Chain A (Chain A aligned by C-alphas)	5CI4 – Water Number in Chain A (Chain A aligned by C-alphas)	5CNS – Water Number in Chain E (Chain E aligned by C-alphas)	5CNS – Water Number in Chain F (Chain F aligned by C-alphas)	Qscore
501	Chain D surface	2001	N/A	N/A	N/A	N/A	N/A	N/A	N/A	0.739029
502	Chain D internal	2066	511	548	525	569	516	N/A	N/A	0.842776
507	Chain D internal	2115	535	545	539	552	558	609	609	0.755458
508	Chain D surface	2167	644	649	671	667	642	N/A	N/A	0.589915
510	Chain C/D interface	2074	679	530	558	532	568	N/A	N/A	0.868074
511	Chain D surface	N/A	645	659	644	642	617	N/A	N/A	0.775572
512	Chain C/D interface	2026	615	653	653	624	593	N/A	N/A	0.79454
513	Chain D internal	2105	569	599	603	592	596	N/A	N/A	0.849179
514	Chain D surface	2127	549	611	565	538	541	N/A	N/A	0.840527
515	Chain D surface	2122	695	694	690	704	694	N/A	N/A	0.60209
516	Chain D surface near Chain B	N/A	578	604	611	576	660	N/A	N/A	0.878622
517	Chain D surface	2169	622	622	555	643	N/A	N/A	N/A	0.774024
518	Chain D surface	2089	N/A	N/A	N/A	N/A	N/A	N/A	N/A	0.781212
519	Chain D surface	2103	564	593	535	570	536	N/A	610	0.752963
520	Chain D surface	2170	N/A	N/A	N/A	N/A	N/A	N/A	N/A	0.617013
521	Chain B/C/D interface	2041	658	648	634	664	632	N/A	N/A	0.785889
522	Chain D surface	2113	681	687	666	647	670	N/A	N/A	0.922288
523	Chain D surface	2112	605	549	592	604	603	N/A	N/A	0.914072
524	Chain D internal	2118	586	597	605	601	610	N/A	N/A	0.742284
525	Chain C/D interface	2079 in Chain A	629	N/A	N/A	589	N/A	N/A	N/A	0.869497
526	Chain D surface	2080	680	670	639	637	625	N/A	N/A	0.804296
527	Chain C/D interface	2104 in Chain A	574	N/A	N/A	N/A	580	N/A	N/A	0.907282
528	Chain D internal	2018	507	567	502	554	515	604	N/A	0.811967

530	Chain D internal	2021	588	645	595	586	577	608	N/A	0.837587
533	Chain C/D interface	2075	596	585	637	623	616	N/A	612	0.855745
534	Chain C/D interface	2020	628	601	601	595	647	N/A	N/A	0.834769
535	Chain B/D interface	N/A	537	634	615	662	681	N/A	N/A	0.767379
536	Chain C/D interface	2064	603	602	622	587	635	N/A	N/A	0.880767
537	Chain C/D interface	2069	602	516	608	607	601	N/A	N/A	0.924188
539	Chain D surface	2152	593	655	654	631	659	N/A	N/A	0.74035
540	Chain D surface	N/A	665	608	618	633	679	N/A	N/A	0.611122
541	Chain D surface	2097	503	603	512	511	511	N/A	N/A	0.801963
542	Chain D surface	N/A	510	561	513	559	549	N/A	N/A	0.849633
544	Chain D surface	2053	522	524	523	509	531	N/A	N/A	0.758194
545	Chain D surface	2136	635	621	640	646	669	N/A	N/A	0.629037
547	Chain C/D interface	2009	686	642	682	691	685	N/A	N/A	0.836917
549	Chain C/D interface	2086	659	644	617	640	673	N/A	N/A	0.670254
551	Chain B/D interface	N/A	570	584	594	635	534	N/A	N/A	0.735835
552	Chain D surface	2161	600	600	578	547	586	N/A	N/A	0.775324
553	Chain C/D interface	N/A	555	628	623	503	645	N/A	N/A	0.871854
554	Chain D surface	2114	N/A	N/A	N/A	N/A	N/A	N/A	N/A	0.817378
555	Chain D internal	2098	N/A	N/A	N/A	N/A	N/A	N/A	N/A	0.631756
556	Chain D surface	2111	546	590	563	598	599	N/A	N/A	0.826425
557	Chain D surface	2015	631	658	663	656	651	N/A	N/A	0.90321
558	Chain D internal	2012	607	652	658	628	609	N/A	N/A	0.871309
559	Chain C/D interface	2043 in Chain A	633	683	675	671	638	N/A	N/A	0.905344
560	Chain D internal	2035	643	650	629	560	566	N/A	N/A	0.814984
561	Chain D surface near Chain C	2005	N/A	N/A	N/A	N/A	N/A	N/A	N/A	0.863626
563	Chain D surface	2100	623	623	626	585	627	N/A	N/A	0.811471

564	Chain C/D interface	2093 in Chain A	N/A	N/A	N/A	N/A	N/A	N/A	N/A	0.711731
565	Chain D near Chain B	N/A	689	666	688	684	608	N/A	N/A	0.78647
567	Chain C/D interface	N/A	505	N/A	528	N/A	N/A	N/A	N/A	0.814825
569	Chain C/D interface	N/A	704	699	699	702	704	N/A	N/A	0.7974
570	Chain D surface near Chain B	N/A	678	505	581	679	710	N/A	N/A	0.683878

*unless the Chain identity is otherwise specified

Table S7. List of waters bound to any chain in the structure found using the "Find Waters" function in Coot (see SI for details) that do not have corresponding water molecules in the crystal structures analyzed above.

Water Number and Chain from "Find Waters" not accounted for by crystal structures	Location	Qscore
1015 in Chain A	Chain A internal	0.834147
936 in Chain B	Chain B/C interface	0.758996
955 in Chain B	Chain B/C interface	0.902583
963 in Chain B	Chain B surface	0.749244
982 in Chain B	Chain B/C interface and Chain B active site	0.912371
984 in Chain B	Chain B active site	0.864741
1037 in Chain B	Chain B surface	0.893012
1038 in Chain B	Chain B surface	0.831865
1076 in Chain B	Chain B/C interface	0.902976
1081 in Chain B	Chain B/D interface	0.925554
1086 in Chain B	Chain B surface	0.84617
1093 in Chain B	Chain B/C interface	0.781017
1102 in Chain B	Chain B/C interface	0.893715
1124 in Chain B	Chain B surface near chain D	0.850708
1156 in Chain B	Chain B/C interface	0.809696
1165 in Chain B	Chain B/C interface	0.823532
1177 in Chain B	Chain B/C interface	0.836857
1178 in Chain B	Chain A/B/C interface	0.860573
509 in Chain C	Chain A/B/C interface	0.750023
549 in Chain C	Chain C internal	0.852757
561 in Chain C	Chain A/C interface	0.877152
572 in Chain C	Chain C/D interface	0.78696
577 in Chain C	Chain B/C interface	0.741394
582 in Chain C	Chain B/C interface	0.866382
589 in Chain C	Chain B/C interface	0.84306
597 in Chain C	Chain C surface	0.805753
503 in Chain D	Chain A/D interface	0.776443
531 in Chain D	Chain D internal	0.881503
546 in Chain D	Chain B/D interface	0.828317

Table S8. List of waters bound to any chain in the structure that do not have corresponding water molecules in the crystal structures analyzed above found by manually walking through the structure.

Water Number and Chain for manually added waters	Location	Qscore
905 in Chain A	Chain A surface	0.61765
913 in Chain A	Chain A surface	0.869532
926 in Chain A	Chain A surface	0.734997
951 in Chain A	Chain A/B interface	0.830304
954 in Chain A	Chain A surface	0.834943
958 in Chain A	Chain A surface	0.84718
965 in Chain A	Chain A/B interface	0.832192
987 in Chain A	Chain A surface	0.730228
995 in Chain A	Chain A surface	0.801112
1014 in Chain A	Chain A surface	0.841313
1019 in Chain A	Chain A surface	0.815642
1033 in Chain A	Chain A surface	0.868972
1034 in Chain A	Chain A surface	0.848711
1040 in Chain A	Chain A surface near dATP site in Chain A	0.721635
1044 in Chain A	Chain A/D interface	0.815237
905 in Chain B	Chain B/C interface	0.647573
915 in Chain B	Chain B/C interface	0.754857
924 in Chain B	Chain B near ATP site	0.402601
926 in Chain B	Chain B/C interface	0.8405
930 in Chain B	Chain B surface	0.915631
932 in Chain B	Chain A/B interface	0.839068
938 in Chain B	Chain B surface	0.654941
948 in Chain B	Chain B/C interface	0.868882
956 in Chain B	Chain B/C interface	0.781581
965 in Chain B	Chain B/C interface	0.875007
977 in Chain B	Chain B/C interface	0.789176
978 in Chain B	Chain B/C interface	0.815292
979 in Chain B	Chain B/C interface	0.800549
981 in Chain B	Chain B surface	0.817583
994 in Chain B	Chain B surface	0.635165
997 in Chain B	Chain B surface	0.794173
1016 in Chain B	Chain B/C interface	0.797765
1018 in Chain B	Chain B/C interface	0.836799
1042 in Chain B	Chain B surface	0.826292
1052 in Chain B	ATP site	0.83499
1055 in Chain B	Chain B/D interface	0.80752

1060 in Chain B	Chain B/C interface	0.810573
1069 in Chain B	Chain B surface	0.727205
1070 in Chain B	Chain B/C interface	0.819343
1071 in Chain B	Chain B surface	0.786581
1083 in Chain B	Chain B/C interface	0.788178
1084 in Chain B	Chain B/C interface	0.860176
1088 in Chain B	Chain B/C interface	0.855077
1103 in Chain B	Chain B surface	0.807777
1114 in Chain B	Chain B/C interface	0.903401
1119 in Chain B	Chain B surface near dATP site in Chain B	0.820286
1132 in Chain B	Chain B surface	0.755591
1134 in Chain B	Chain B/C interface	0.808806
1141 in Chain B	Chain B/C interface	0.788936
1147 in Chain B	Chain B/C interface	0.800612
1150 in Chain B	Chain B/C interface	0.75509
1151 in Chain B	Chain B surface	0.792592
1154 in Chain B	Chain B surface near dATP site in Chain B	0.779656
1155 in Chain B	Chain B surface	0.835391
1162 in Chain B	Chain B surface	0.754283
1164 in Chain B	Chain A/B surface	0.857396
1168 in Chain B	Chain B surface	0.811115
1174 in Chain B	Chain B surface	0.765418
1186 in Chain B	Chain B surface near dATP site in Chain B	0.748357
1187 in Chain B	Chain B surface near Chain C	0.742427
1190 in Chain B	Chain A/B/C interface	0.808241
1191 in Chain B	Chain B surface	0.788587
502 in Chain C	Chain B/C interface	0.766246
511 in Chain C	Fe_2O site	0.544792
513 in Chain C	Chain B/C interface	0.813343
519 in Chain C	Chain B/C interface	0.823887
520 in Chain C	Chain C/D interface	0.626192
521 in Chain C	Chain B/C interface	0.760901
535 in Chain C	Chain B/C interface	0.845528
539 in Chain C	Chain A/C interface	0.732206
540 in Chain C	Chain C surface	0.794983
542 in Chain C	Chain B/C interface	0.832889
543 in Chain C	Chain B/C interface	0.763121
550 in Chain C	Chain C surface	0.798474

556 in Chain C	Chain B/C interface	0.897779
562 in Chain C	Chain B/C interface	0.746886
563 in Chain C	Chain B/C/D interface	0.736722
565 in Chain C	Chain B/C interface	0.886891
567 in Chain C	Chain B/C interface	0.859275
570 in Chain C	Chain C/D interface	0.882411
578 in Chain C	Chain C surface	0.875736
584 in Chain C	Chain B/C interface	0.87105
586 in Chain C	Chain B/C interface	0.76788
592 in Chain C	Chain A/C interface	0.744232
596 in Chain C	Chain A/C interface	0.881656
599 in Chain C	Chain B/C interface	0.804872
600 in Chain C	Chain A/B/C interface	0.808845
602 in Chain C	Chain A/B/C interface	0.800009
605 in Chain C	Chain B/C interface	0.891546
606 in Chain C	Chain C surface	0.812837
607 in Chain C	Chain A/B/C interface	0.79152
504 in Chain D	Fe_2O site	0.669508
505 in Chain D	Chain C/D interface	0.684958
509 in Chain D	Chain A/D interface	0.803428
529 in Chain D	Chain D surface	0.555955
532 in Chain D	Chain D surface near Chain B	0.841204
538 in Chain D	Chain A/D interface	0.831821
543 in Chain D	Chain B/C/D interface	0.793863
548 in Chain D	Chain D surface near Chain B	0.892428
562 in Chain D	Chain B/D interface near Chain C	0.739774
566 in Chain D	Chain D surface	0.698096
568 in Chain D	Chain B/D interface near Chain C	0.773393

Table S9. Direct interactions across β′–α, β–α′, β′–α′ subunit interfaces. Distances less than or equal to 3.5 Å between key residues in the indicated subunits.

β'-α Interactions	
Residues	*Distance (Å)*
K229β' (NZ) – E397α (OE1)	3.4
K229β' (NZ) – E397α (OE2)	2.5
β-α' Interactions	
N131β – E314α′ (backbone O)	3.2
R127β – N322α′ (backbone O)	2.8
R127β – R323α′ (backbone O)	3.3
R127β (backbone O) – N328α′	3.3
S134β – R323α′	2.9
β'-α' Interactions	
D362β′ (backbone N) – V709α′ (backbone O)	3.2
D366β′ (backbone N) – Q712α′	3.1
D29β′ – R323α′ (NE)	3.3
D29β′ – R323α′ (NH2)	3.4
Q30β′ – R323α′	2.7
Q360β′ (backbone O) – V709α′ (backbone N)	3.3
D362β′ (backbone N) – V709α′ (backbone O)	3.2
D362β′ (backbone O) – M711α′ (backbone N)	2.5
D368β′ (OD1) – Q712α′	2.5
D368β′ (OD2) – Q712α′	3.4

Table S10. Direct interactions across the α–β interface. Distances less than or equal to 3.5 Å between key residues in the β and α subunits. β-C-terminus interactions not observed in the previously published $\alpha2\beta2$ structure are bolded. Note that not all observed interactions were reported in **Table S2** of the previous publication.

β-C-Terminus	
Residues	*Distance (Å)*
**D342β (backbone O) – W670α**	3.3
D342β (OD1) – R735α (NE)	2.6
D342β (OD2) – R735α (NH2)	3.0
D342β (OD1) – R735α (NH2)	3.4
N343β (backbone O)– I644α (backbone N)	3.0
N343β – V642α (backbone O)	2.8
Q345β (backbone N) – I644α (backbone O)	2.6
Q345β (backbone O) – A646α (backbone N)	3.5
**Q345β – E623α**	2.5
A347β (backbone N) – A646α (backbone O)	3.0
**P348β (backbone O) – R411α** (NH1)	2.5
**P348β (backbone O) – R411α** (NH2)	3.1
**Q349β – R411α**	3.4
**Q349β – Y731α**	3.1
Q349β – Y730α	3.4
Q349β – D334α	3.1
E350β – K154α	3.1
**E350β – T624α**	3.4
**S355β – T734α (backbone O)**	2.8
Q360β – G707α (backbone O)	3.4
**Q360β (backbone O) – V709α (backbone N)**	3.1
D362β (backbone N) – V709α (backbone O)	2.8
**D362β – K708α**	3.5
D366β (backbone N) – Q712α	2.8
D366β (backbone O) – Q712α	3.3
**L375β (backbone O) – K584α**	2.4
Observed Previously (PDB 6W4X) but Not Present in Current Structure	
Residues	*Distance in Current Structure (Å) (Distance in Previous Structure (Å))*
Q349β – Y413α	3.7 (3.2)
E350β – S647α	4.2 (2.4)
Other Region	
Residues	*Distance (Å)*
K42β – D736α (backbone O)	3.3

R49β – N322α (backbone O)	3.2
R49β – G324α (backbone O)	2.7
E52β – N322α	2.8
E220β – K64α	3.1
R236β – D736α	3.5
N300β – I47α (backbone O)	3.0
Y307β – Q742α	2.7
Y310β – D743α (backbone O)	2.2
Y310β – D743α (backbone N)	3.0
R315β (NH1) – D740α (OD1)	3.3
R315β (NH2) – D740α (OD2)	2.9
N330β – Q742α (backbone O)	3.0
N336β – Q742α (backbone O)	2.6
N336β – Q742α (backbone N)	2.8
L339β (backbone O) – D740α (backbone N)	3.3
S341β (backbone N) – A738α (backbone O)	3.3
S341β – D736α	2.7

Table S11. Water-bridged interactions across the α–β interface. Waters newly identified in this structure are bolded.

β-C-Terminus	
Residues	*Bridging Water Number and Chain*
E350β, SN-CDPα	1080 in Chain B
E350β, G299α (backbone O), SN-CDPα	**982 in Chain B**
S350β (backbone O), D736α	**955 in Chain B**
G359β (backbone N), Q404α	573 in Chain C
G349β, R411α, D334α	503 in Chain C
V346β (backbone O), R639α	**562 in Chain C**
E352β, K320α	1120 in Chain B
Q345β, R639α (backbone O)	1019 in Chain B
S355β, T734α (backbone O)	558 in Chain C
E350β, T624α	1056 in Chain B
D342β (backbone O), V344β (backbone O), W670α, R639α	537 in Chain C
S363β, S400α	1110 in Chain B
E350β, D649α	**502 in Chain C**
E364β (backbone O, N), M711α (backbone N)	506 in Chain C
Q345β, E623α	**1141 in Chain B**
D342β, D736α, D737α (backbone N)	**1134 in Chain B**
E352β (backbone N), E225β, K648α	**521 in Chain C**
Other Region	
Residues	*Bridging Water Number*
D226β (backbone N), E225β (backbone N), D58β, K648α	1057 in Chain B
M296β (backbone O), S295β, Q48α (backbone O)	545 in Chain C
E220β (backbone O), I651α (backbone O)	576 in Chain C
R57β, V297α (backbone O)	**519 in Chain C**
P333β (backbone O), R653α	**1147 in Chain B**
R315β, A741α (backbone O)	**905 in Chain B**

Table S12. Water-bridged interactions across β′–α, β–α′, β′–α′ subunit interfaces. Waters newly identified in this structure are bolded.

β′-α Interactions	
Residues	*Bridging Water Number and Chain*
E52β′, Q404α	1032 in Chain B
F47β′ (backbone O), R323α	**546 in Chain D**
E52β′ (backbone O), Q404α	**543 in Chain D**
β-α′ Interactions	
R59(C)β, E312α′, T276α′ (backbone O), C278α′ (backbone N), I279α′ (backbone N)	962 in Chain A
R59(B)β, N328α′ (backbone O), S315α′ (backbone O), S315α′	967 in Chain A
R59(C)β, S315α′, E312α′	972 in Chain A
R59(B)β, N131β, R329α′	526 in Chain C
β′-α′ Interactions	
D362β′, D362β′ (backbone O), E364β′ (backbone O), E364β′ (backbone N), M711α′ (backbone N), Q712α′ (backbone N)	**503 in Chain D**
Q360β′, A407α′ (backbone O)	550 in Chain D
D29β′ (backbone O), R323α′	**1044 in Chain A**

Table S13. List of PCET pathway interactions with water molecules in α and β. Interaction distances are listed and waters newly identified in this structure are bolded.

Interacting Residue or Water	Water Number and Chain	Distance (Å)
Fe_2O (FE1)	**511 in Chain C**	2.0
Y122β (OH)	**511 in Chain C**	3.2
D237β (OD1)	517 in Chain C	2.4
R236β (NE)	517 in Chain C	2.3
R236β (NH2)	517 in Chain C	2.8
R236β (NH2)	536 in Chain C	3.5
Y356β (backbone O)	536 in Chain C	2.7
Water 26	536 in Chain C	3.2
E52β (OE2)	546 in Chain C	2.7
**Water 1150 in Chain B**	546 in Chain C	3.4
V353β (backbone O)	516 in Chain C	2.3
E52β (OE2)	516 in Chain C	2.9
Y356β (OH)	507 in Chain C	2.2
N322α (ND2)	**1150 in Chain B**	3.3
E52β (backbone O)	**582 in Chain C**	3.2
**Water 936 in Chain B**	**582 in Chain C**	2.3
**Water 1088 in Chain B**	**936 in Chain B**	3.5
Water 594 in Chain C	**1088 in Chain B**	2.9
V353β (backbone N)	594 in Chain C	3.4
Y731α (OH)	1014 in Chain B	2.7
E623α (OE2)	1014 in Chain B	3.8
Water 1027 in Chain B	1014 in Chain B	3.0
C439α (SG)	1027 in Chain B	3.0
SN-CDP phosphate group (O1A)	1027 in Chain B	2.7
SN-CDP base (N4)	**982 in Chain B**	2.5
Q349β (backbone O)	**982 in Chain B**	2.7
SN-CDP base (N3)	**982 in Chain B**	3.5
Water 1080 in Chain B	564 in Chain C	2.6
Q349β (backbone N)	564 in Chain C	2.9
Water 1014 in Chain B	564 in Chain C	2.9
SN-CDP base (N4)	1080 in Chain B	2.9
Water 918 in Chain B	1080 in Chain B	3.1
SN-CDP phosphate group (O1B)	918 in Chain B	2.3
SN-CDP phosphate group (O2B)	918 in Chain B	2.2
SN-CDP phosphate group (O3A)	**984 in Chain B**	3.1

SN-CDP ribose oxygen (O4')	**984 in Chain B**	2.8
SN-CDP phosphate group (O3A)	992 in Chain B	2.6
SN-CDP phosphate group (O2A)	992 in Chain B	3.2

Table S14. List of PDB IDs for crystal structures used to identify previously observed water structures within the current cryo-EM density.

PDB ID	Resolution (Å)	Subunit
1R1R	2.90	$\alpha2$
2X0X	2.30	$\alpha2$
2XAP	2.10	$\alpha2$
2XAX	2.75	$\alpha2$
2XAY	2.65	$\alpha2$
2XAZ	2.60	$\alpha2$
2XO4	2.50	$\alpha2$
2XO5	2.70	$\alpha2$
5CNS	2.98	$\alpha2$
8VHN	2.62	$\alpha2$
2XOF	2.20	$\beta2$
5CI0	2.25	$\beta2$
5CI1	1.95	$\beta2$
5CI2	2.25	$\beta2$
5CI3	2.40	$\beta2$
5CI4	2.05	$\beta2$

Table S15. Additional hydrogen bonding interactions by PCET waters not depicted in Figure S14. Interaction distances are listed and waters newly identified in this structure are bolded.

Water Number and Chain	Interacting Residue or Water	Distance (Å)
**Water 511 in Chain C**	E238β	2.5
**Water 511 in Chain C**	D84β	2.6
Water 517 in Chain C	L233β (backbone O)	3.1
Water 517 in Chain C	F46β (backbone O)	3.5
Water 536 in Chain C	F47β (backbone O)	2.6
Water 546 in Chain C	N/A	N/A
**Water 1150 in Chain B**	S408α (backbone O)	3.4
**Water 1150 in Chain B**	Water 1140 in Chain B	2.3
**Water 936 in Chain B**	K320α (backbone O)	2.3
**Water 1088 in Chain B**	T409α (backbone O)	3.0
Water 594 in Chain C	Water 1120 in Chain B	3.3
Water 507 in Chain C	N/A	N/A
Water 1104 in Chain B	Water 1115 in Chain B	2.2
Water 1104 in Chain B	T734α (backbone N)	3.0
Water 1104 in Chain B	S355β (backbone O)	3.3
Water 1014 in Chain B	N/A	N/A
Water 1080 in Chain B	Q350β	3.1
Water 918 in Chain B	T624α (backbone N)	3.3
Water 918 in Chain B	R298α	3.5
Water 918 in Chain B	T624α	2.7
Water 918 in Chain B	SN-CDP phosphate group (O1A)	3.0
Water 1027 in Chain B	N/A	N/A
**Water 982 in Chain B**	G299α (backbone O)	2.7
**Water 984 in Chain B**	S224α	2.3
Water 992 in Chain B	S625α	3.5
Water 992 in Chain B	Water 1179 in Chain B	2.7

Table S16. List of waters found within the two water channels. Interaction distances are listed and waters newly identified in this structure are bolded.

Yellow Channel		
**Waters located within channel**	**Interacting Residue or Water**	**Distance (Å)**
Water 546 in Chain C	Water 536 in Chain C	3.2
Water 546 in Chain C	**Water 1150 in Chain B**	3.4
Water 546 in Chain C	E52β	2.7
Water 536 in Chain C	F47β (backbone O)	2.6
Water 536 in Chain C	Y356β (backbone O)	2.7
Water 536 in Chain C	R236β	3.5
Water 1140 in Chain B	**Water 1150 in Chain B**	2.3
Water 1140 in Chain B	S408α (backbone O)	3.2
Water 1140 in Chain B	S408α	3.5
Water 1140 in Chain B	R323α	3.3
Water 1140 in Chain B	N322α (OD1)	3.4
Water 1140 in Chain B	N322α (ND2)	3.5
Water 1140 in Chain B	N322α (backbone N)	3.5
Water 1109 in Chain B	Water 1006 in Chain A	3.5
Water 1109 in Chain B	Water 580 in Chain C	2.1
Water 1109 in Chain B	K290α (backbone O)	3.1
Water 1006 in Chain A	**Water 1178 in Chain B**	2.6
Water 1006 in Chain A	Water 1109 in Chain B	3.5
Water 1006 in Chain A	K290α (backbone O)	3.2
Water 1006 in Chain A	S291α (backbone O)	3.2
Water 1006 in Chain A	T276α'	3.0
**Water 582 in Chain C**	**Water 936 in Chain B**	2.3
**Water 582 in Chain C**	E52β (backbone O)	3.2
**Water 1178 in Chain B**	**Water 1038 in Chain B**	2.5
**Water 1178 in Chain B**	Water 603 in Chain C	3.1
**Water 1178 in Chain B**	Water 1182 in Chain B	2.4
Water 591 in Chain C	Q349β (backbone O)	3.3
**Water 1038 in Chain B**	**Water 1178 in Chain B**	2.5
**Water 1038 in Chain B**	C292α (backbone O)	2.8
**Water 1038 in Chain B**	G300α (backbone N)	3.0
Water 594 in Chain C	Water 1120 in Chain B	3.3
Water 594 in Chain C	**Water 1088 in Chain B**	2.9
Water 594 in Chain C	V353β (backbone N)	3.4
**Water 936 in Chain B**	**Water 1088 in Chain B**	3.5
**Water 936 in Chain B**	K320α	2.3

Water 603 in Chain C	Water 1182 in Chain B	3.4
Water 603 in Chain C	**Water 602 in Chain C**	3.3
Water 1120 in Chain B	Water 958 in Chain B	3.3
Water 1120 in Chain B	E352β	3.4
Water 1120 in Chain B	K320α	3.1
Water 1040 in Chain B	K290α	2.8
Water 1182 in Chain B	**Water 584 in Chain C**	3.2
Water 1182 in Chain B	**Water 602 in Chain C**	3.0
Water 1182 in Chain B	**Water 600 in Chain C**	2.3
**Water 584 in Chain C**	R57β	3.2
Water 958 in Chain B	Water 503 in Chain C	3.3
Water 958 in Chain B	R411α (NE)	3.3
Water 958 in Chain B	R411α (NH2)	2.4
Water 958 in Chain B	D334α	2.9
Water 580 in Chain C	S56β	3.2
**Water 1150 in Chain B**	S408α (backbone O)	3.4
**Water 1150 in Chain B**	N322α	3.3
**Water 1088 in Chain B**	T409α (backbone O)	3.0
**Water 567 in Chain C**	**Water 543 in Chain C**	2.4
**Water 567 in Chain C**	P348β (backbone O)	3.0
**Water 602 in Chain C**	See previous entries	See previous entries
**Water 600 in Chain C**	**Water 519 in Chain C**	3.3
**Water 513 in Chain C**	R57β (backbone N)	3.4
**Water 513 in Chain C**	S56β	3.2
**Water 513 in Chain C**	D54β	2.3
**Water 513 in Chain C**	D54β (backbone O)	3.4
**Water 543 in Chain C**	Q349β (backbone O)	2.7
Water 516 in Chain C	V353β (backbone O)	2.3
Water 516 in Chain C	E52β	2.9
**Pink Channel**		
**Waters located within channel**	**Interacting Residue or Water**	**Distance (Å)**
Water 507 in Chain C	Y356β	2.2
**Water 1093 in Chain B**	Water 1115 in Chain B	3.1
**Water 1093 in Chain B**	**Water 577 in Chain C**	2.9
**Water 1093 in Chain B**	**Water 562 in Chain C**	2.3
**Water 1093 in Chain B**	R639α (NH1)	3.1
**Water 1093 in Chain B**	R639α (NH2)	3.0
**Water 577 in Chain C**	**Water 586 in Chain C**	2.3
**Water 577 in Chain C**	V344β (backbone O)	3.1

**Water 562 in Chain C**	Water 1129 in Chain B	2.6
**Water 562 in Chain C**	R639α	3.4
**Water 562 in Chain C**	V346β (backbone O)	2.9
Water 558 in Chain C	Water 537 in Chain C	3.5
Water 558 in Chain C	**Water 1134 in Chain B**	2.4
Water 558 in Chain C	T734α (backbone O)	3.4
Water 558 in Chain C	S355β	3.0
Water 558 in Chain C	S341β (backbone O)	2.9
Water 537 in Chain C	W670α	3.2
Water 537 in Chain C	R639α	2.9
Water 537 in Chain C	V344β (backbone O)	3.4
Water 537 in Chain C	D342β (backbone O)	2.6
**Water 1134 in Chain B**	G737α (backbone N)	3.4
**Water 1134 in Chain B**	D736α	3.2
**Water 1134 in Chain B**	D342β	3.4
**Water 1134 in Chain B**	S341β (backbone N)	3.4
**Water 1134 in Chain B**	S341β	3.3
**Water 1114 in Chain B**	A738α (backbone O)	3.1
**Water 1114 in Chain B**	D342β (backbone N)	3.3
**Water 586 in Chain C**	S354β (backbone N)	3.2

SI References

1. S. P. Salowe, J. Stubbe, Cloning, overproduction, and purification of the B2 subunit of ribonucleoside-diphosphate reductase. *J.Bacteriol.* **165 (2)**, 363-366 (1986).
2. S. P. Salowe, M. A. Ator, J. Stubbe, Products of the inactivation of ribonucleoside diphosphate reductase from *Escherichia coli* with 2'-azido-2'-deoxyuridine 5'-diphosphate. *Biochemistry* **26 (12)**, 3408-3416 (1987).
3. N. Ando, E. J. Brignole, C. M. Zimanyi, M. A. Funk, K. Yokoyama, F. J. Asturias, J. Stubbe, C. L. Drennan, Structural interconversions modulate activity of *Escherichia coli* ribonucleotide reductase. *Proc. Natl. Acad. Sci. U.S.A.* **108**, 21046-21051 (2011).
4. A. L. Persson, M. Eriksson, B. Katterle, S. Pötsch, M. Sahlin, B. M. Sjöberg, A new mechanism-based radical intermediate in a mutant R1 protein affecting the catalytically essential Glu441 in *Escherichia coli* ribonucleotide reductase. *J. Biol. Chem.* **272**, 31533-31541 (1997).
5. S. Q. Zheng, E. Palovcak, J.-P. Armache, K. A. Verba, Y. Cheng, D. A. Agard, MotionCor2: anisotropic correction of beam-induced motion for improved cryo-electron microscopy. *Nat. Methods* **14**, 331-332 (2017).
6. D. Kimanius, L. Dong, G. Sharov, T. Nakane, S. H. W. Scheres, New tools for automated cryo-EM single-particle analysis in RELION-4.0. *Biochem. J.* **478**, 4169-4185 (2021).
7. A. Rohou, N. Grigorieff, CTFFIND4: Fast and accurate defocus estimation from electron micrographs. *J. Struct. Biol.* **192**, 216-221 (2015).
8. T. Bepler, A. Morin, M. Rapp, J. Brasch, L. Shapiro, A. J. Noble, B. Berger, Positive-unlabeled convolutional neural networks for particle picking in cryo-electron micrographs. *Nat. Methods* **16**, 1153-1160 (2019).
9. P. D. Adams, P. V. Afonine, G. Bunkoczi, V. B. Chen, I. W. Davis, N. Echols, J. J. Headd, L. W. Hung, G. J. Kapral, R. W. Grosse-Kunstleve, A. J. McCoy, N. W. Moriarty, R. Oeffner, R. J. Read, D. C. Richardson, J. S. Richardson, T. C. Terwilliger, P. H. Zwart, PHENIX: a comprehensive Python-based system for macromolecular structure solution. *Acta Crystallogr. Sect. D* **66**, 213-221 (2010).
10. G. Kang, A. T. Taguchi, J. Stubbe, C. L. Drennan, Structure of a trapped radical transfer pathway within a ribonucleotide reductase holocomplex. *Science* **368**, 424-427 (2020).
11. T. D. Goddard, C. C. Huang, E. C. Meng, E. F. Pettersen, G. S. Couch, J. H. Morris, T. E. Ferrin, UCSF ChimeraX: Meeting modern challenges in visualization and analysis. *Protein Sci.* **27**, 14-25 (2018).
12. C. J. Williams, J. J. Headd, N. A.-O. Moriarty, M. G. Prisant, L. L. Videau, L. N. Deis, V. Verma, D. A. Keedy, B. J. Hintze, V. B. Chen, S. Jain, S. M. Lewis, W. B. Arendall, 3rd, J. Snoeyink, P. D. Adams, S. C. Lovell, J. S. Richardson, D. C. Richardson, MolProbity: More and better reference data for improved all-atom structure validation. *Protein Sci.* **27**, 293-315 (2018).
13. A. Morin, B. Eisenbraun, J. Key, P. C. Sanschagrin, M. A. Timony, M. Ottaviano, P. Sliz, Cutting edge: Collaboration gets the most out of software. *eLife* **2**, 6 (2013).
14. J. Fritscher, E. Artin, S. Wnuk, G. Bar, J. H. Robblee, S. Kacprzak, M. Kaupp, R. G. Griffin, M. Bennati, J. Stubbe, Structure of the nitrogen-centered radical

formed during inactivation of *E. coli* ribonucleotide reductase by 2‘-azido-2‘-deoxyuridine-5‘-diphosphate: Trapping of the 3‘-ketonucleotide. *J. Am. Chem. Soc.* **127**, 7729-7738 (2005).
15. S. Salowe, J. M. Bollinger, M. Ator, J. Stubbe, J. McCracken, J. Peisach, M. C. Samano, M. J. Robins, Alternative model for mechanism-based inhibition of *Escherichia coli* ribonucleotide reductase by 2'-azido-2'-deoxyuridine 5'-diphosphate. *Biochemistry* **32**, 12749-12760 (1993).
16. W. A. van der Donk, J. Stubbe, G. J. Gerfen, B. F. Bellew, R. G. Griffin, EPR investigations of the inactivation of *E. coli* ribonucleotide reductase with 2’-azido-2'-deoxyuridine 5’-diphosphate: Evidence for the involvement of the thiyl radical of C225-R1. *J. Am. Chem. Soc.* **117**, 8908-8916 (1995).
17. P. Emsley, B. Lohkamp, W. G. Scott, K. Cowtan, Features and development of Coot. *Acta Crystallogr. Sect. D* **66**, 486-501 (2010).
18. J. Gu, T. Liu, R. Guo, L. Zhang, M. Yang, The coupling mechanism of mammalian mitochondrial complex I. *Nat. Struct. Mol. Biol.* **29**, 172-182 (2022).
19. K. Yokoyama, U. Uhlin, J. Stubbe, Site-specific incorporation of 3-nitrotyrosine as a probe of pKa perturbation of redox-active tyrosines in ribonucleotide reductase. *J. Am. Chem. Soc.* **132**, 8385-8397 (2010).
20. M. Eriksson, U. Uhlin, S. Ramaswamy, M. Ekberg, K. Regnström, B.-M. Sjöberg, H. Eklund, Binding of allosteric effectors to ribonucleotide reductase protein R1: reduction of active-site cysteines promotes substrate binding. *Structure* **5**, 1077-1092 (1997).
21. E. C. Minnihan, M. R. Seyedsayamdost, U. Uhlin, J. Stubbe, Kinetics of radical intermediate formation and deoxynucleotide production in 3-aminotyrosine-substituted *Escherichia coli* ribonucleotide reductases. *J. Am. Chem. Soc.* **133**, 9430-9440 (2011).
22. C. M. Zimanyi, P. Y.-T. Chen, G. Kang, M. A. Funk, C. L. Drennan, Molecular basis for allosteric specificity regulation in class Ia ribonucleotide reductase from *Escherichia coli*. *eLife* **5**, e07141 (2016).
23. M. A. Funk, C. M. Zimanyi, G. A. Andree, A. E. Hamilos, C. L. Drennan, How ATP and dATP act as molecular switches to regulate enzymatic activity in the prototypical bacterial class Ia ribonucleotide reductase. *Biochemistry* 10.1021/acs.biochem.4c00329 (2024).
24. K. Yokoyama, U. Uhlin, J. Stubbe, A hot oxidant, 3-NO_2Y122 radical, unmasks conformational gating in ribonucleotide reductase. *J. Am. Chem. Soc.* **132**, 15368-15379 (2010).
25. P. H. Oyala, K. R. Ravichandran, M. A. Funk, P. A. Stucky, T. A. Stich, C. L. Drennan, R. D. Britt, J. Stubbe, Biophysical characterization of fluorotyrosine probes site-specifically incorporated into enzymes: *E. coli* ribonucleotide reductase as an example. *J. Am. Chem. Soc.* **138**, 7951-7964 (2016).
26. G. Pintilie, K. Zhang, Z. Su, S. Li, M. F. Schmid, W. Chiu, Measurement of atom resolvability in cryo-EM maps with Q-scores. *Nat. Methods* **17**, 328-334 (2020).
27. E. Chovancova, A. Pavelka, P. Benes, O. Strnad, J. Brezovsky, B. Kozlikova, A. Gora, V. Sustr, M. Klvana, P. Medek, L. Biedermannova, J. Sochor, J. Damborsky, CAVER 3.0: A tool for the analysis of transport pathways in dynamic protein structures. *PLoS Comp. Biol.* **8**, e1002708 (2012).

28. A.-J. Li, R. Nussinov, A set of van der Waals and coulombic radii of protein atoms for molecular and solvent-accessible surface calculation, packing evaluation, and docking. *Proteins: Struct., Funct., Bioinf.* **32**, 111-127 (1998).
29. G. A. Jeffrey, *An Introduction to Hydrogen Bonding (Topics in Physical Chemistry)* (Oxford University Press, 1997).
30. E. Krissinel, K. Henrick, Inference of macromolecular assemblies from crystalline state. *J. Mol. Biol.* **372**, 774-797 (2007).
